# Phylogeny of Plant CAMTAs and Role of AtCAMTAs in Nonhost Resistance to *Xanthomonas oryzae* pv. *oryzae*

**DOI:** 10.3389/fpls.2016.00177

**Published:** 2016-02-29

**Authors:** Hafizur Rahman, Juan Yang, You-Ping Xu, Jean-Pierre Munyampundu, Xin-Zhong Cai

**Affiliations:** ^1^Institute of Biotechnology, College of Agriculture and Biotechnology, Zhejiang UniversityHangzhou, China; ^2^Center of Analysis and Measurement, Zhejiang UniversityHangzhou, China; ^3^State Key Laboratory of Rice Biology, Zhejiang UniversityHangzhou, China

**Keywords:** CAMTA, phylogeny, protein interaction network, nonhost resistance, *Xanthomonas oryzae* pv. *oryzae*

## Abstract

Calmodulin-binding transcription activator (CAMTA) constitutes one of the most important Ca^2+^/CaM-regulated transcription factor families in plants. Nevertheless, the phylogeny, protein interaction network, and role in nonhost resistance of plant CAMTAs are not well understood. In this study, 200 *CAMTA* genes were identified from 35 species representing four major plant lineages. The *CAMTA* genes were conserved in multicellular land plants but absent in unicellular eukaryotes, and were likely to emerge from the fusion of two separate genes encoding a CAMTA-like protein and an IQ/CaM binding motif containing protein, respectively, in the embryophyta lineage ancestor. Approximately one fourth of plant CAMTAs did not contain a TIG domain. This non-TIG class of CAMTAs seems to have newly evolved through mutation of some key amino acids in the TIG domain of flowering land plants after divergence from the non-flowering plants. Phylogenetic analysis classified CAMTA proteins into three major groups and nine distinct subgroups, a result supported by protein domain and motif conservation analyses. Most (59.0 and 21.5%) of the identified *CAMTA* genes contained 12 or 11 introns, respectively. Gene duplication, intron invasion, enlargement and turnover, as well as exon rearrangements and skipping have apparently occurred during evolution of the CAMTA family. Moreover, 38 potential interactors of six Arabidopsis CAMTAs were predicted and 10 predicted target genes of *AtCAMTA3* exhibited changes in expression between *Atcamta3* mutants and wild-type plants. The majority of predicted interactors are transcription factors and/or Ca^2+^/CaM-regulated proteins, suggesting that transcriptional regulation of the target genes might be the dominant functional mechanism of AtCAMTAs, and AtCAMTAs might act together with other Ca^2+^ signaling components to regulate Ca^2+^-related biological processes. Furthermore, functional analyses employing *Atcamta* mutants revealed that *AtCAMTA3* negatively regulated the immunity triggered by flg22 and nonhost resistance to *Xanthomonas oryzae* pv. *oryzae* via repressing accumulation of reactive oxygen species probably by targeting *CBP60G, EDS1*, and *NDR1* and involving SA pathway.

## Introduction

Calcium is a universal secondary messenger used by plants to coordinate their responses to a wide range of biotic and abiotic stresses (Reddy et al., 2011). As a major Ca^2+^ sensor protein, calmodulin (CaM) can bind to certain transcription factors (Onions et al., 2000; Bouché et al., 2002) and is involved in plant development, plant-microbe interactions, and stress responses (Du and Poovaiah, 2005; Gleason et al., 2006; Du et al., 2011; Zhao et al., 2013). Calmodulin-binding transcription activators (CAMTAs), also referred to as signal-responsive proteins (SR) or ethylene-induced CaM binding proteins, were first discovered in plants (NtER1) in a screen for CaM binding proteins (Reddy et al., 2000; Yang and Poovaiah, 2000, 2002; Bouché et al., 2002). Follow-up studies showed that CAMTAs belong to a conserved transcription factor (TF) family that exists in all the examined multicellular eukaryotes (Reddy et al., 2000; Bouché et al., 2002; Yang and Poovaiah, 2002; Finkler et al., 2007). This family of TFs possesses multiple domains generally including a CG-1 domain, a TIG domain, an ankyrin (ANK) repeat domain, an IQ domain, and a CaM binding (CaMB) domain that are located in turn from the N terminus to the C terminus. These domains confer different functions. The CG-1 domain is a substrate-specific DNA binding domain. The TIG (Transcription-associated Immuno Globulin domain/Immunoglobulin-like fold shared by plexins and transcription factors) domain is involved in non-specific DNA binding. The ANK domain plays a role in protein–protein interaction. The IQ domain interacts with CaM in a Ca^2+^-independent manner, while the CaMB domain binds CaM in a Ca^2+^-dependent way (Bouché et al., 2002; Yang and Poovaiah, 2002; Finkler et al., 2007; Du et al., 2009; Yang et al., 2012). Notably, some CAMTAs do not carry a TIG domain. How universal this non-TIG type of CAMTAs exists remains unknown.

The biological functions of CAMTAs are being revealed but mainly only in Arabidopsis and tomato. In Arabidopsis, six *CAMTA* genes differentially respond to a variety of external signals, such as cold, wounding and drought, as well as hormonal signals like ethylene and ABA (Reddy et al., 2000; Yang and Poovaiah, 2002). The functions of AtSRs/CaMTAs were found to be dependent on their interaction with Ca^2+^/CaM (Choi et al., 2005; Du et al., 2009). The knockout of *AtCAMTA3* leads to increased accumulation of salicylic acid (SA) and enhanced disease resistance to both bacterial (Du et al., 2009) and fungal pathogens (Nie et al., 2012) but reduced resistance against insect herbivores (Laluk et al., 2012; Qiu et al., 2012). Similarly, one rice *CAMTA* mutant (*oscbt-1*) exhibits enhanced resistance to blast fungal pathogen and leaf blight bacterial pathogen (Koo et al., 2009). *CAMTA1* and *CAMTA3* are also important for plant tolerance to low temperature and freezing tolerance (Kim et al., 2013) and knockout of those genes significantly reduces cold tolerance (Doherty et al., 2009). While in tomato, seven CAMTAs have been identified. They are differentially expressed in tomato tissues and during fruit development and ripening and respond to biotic and abiotic stimuli (Yang et al., 2012, 2013). Silencing of two tomato CAMTAs SlSR1 and SlSR3L enhances resistance to bacterial and fungal pathogens while silencing of SlSR1L reduces resistance to drought stress tolerance (Li et al., 2014). As for the functional mechanism, it has been revealed that CAMTAs bind to a 6-bp CGCG *cis*-element (A/C/G)CGCG(C/G/T) of the targeting gene promoter and thereby regulate the expression of the target genes (Yang and Poovaiah, 2002; Kaplan et al., 2007; Walley et al., 2007; Du et al., 2009; Nie et al., 2012). Nevertheless, role of CAMTAs in plant nonhost resistance; a type of strong, broad-spectrum, and durable resistance to non-adapted pathogens (Senthil-Kumar and Mysore, 2013), remains unclear.

To date, genome-wide identification of CAMTA family has been performed in Arabidopsis (Bouché et al., 2002), rice (Choi et al., 2005; Koo et al., 2009), tomato (Yang et al., 2012), grape (Shangguan et al., 2014), soybean (Wang et al., 2015), *M. truncatula* (Yang et al., 2015), and maize (Yue et al., 2015). However, comprehensive analyses of CAMTA proteins from a variety of plant species at diverse phylogenetic locations are still lacking. To understand the origin, phylogeny, and structural evolution of plant *CAMTA* genes, we systemically identified the *CAMTA* gene family in 35 genome-completed land plant species, ranging from moss to flowering plants. We demonstrated that plant CAMTAs were likely to emerge from the fusion of two separate genes encoding a CAMTA-like protein and an IQ/CaM binding motif containing protein, respectively, in the embryophyta lineage ancestor, and that non-TIG class of CAMTAs evolved recently in flowering plant species. Moreover, our investigation on the protein interaction network of CAMTA family in Arabidopsis revealed that transcriptional regulation of the target genes might be the dominant functional mechanism of AtCAMTAs. Furthermore, to understand the function of *CAMTA* genes in plant disease resistance, we analyzed the involvement of Arabidopsis *CAMTAs* in nonhost resistance to the agriculturally important bacterial pathogen *Xanthomonas oryzae* pv. *oryzae* (*Xoo*). We revealed that *AtCAMTA3* negatively regulates this nonhost resistance via altering accumulation of reactive oxygen species (ROS). Our results provide insights into the phylogeny and function of *CAMTA* genes in plants.

## Materials and methods

### Identification of CAMTA proteins in plants

A BLASTP search was performed against fully sequenced genomes of green plants in Phytozome (http://www.Phytozome.net) and NCBI (http://www.ncbi.nlm.nih.gov/) using Arabidopsis and tomato CAMTA proteins as queries. All retrieved non-redundant sequences were collected and subjected to domain analysis using the Pfam (http://pfam.sanger.ac.uk/) and Conserved Domain Database (CDD, http://www.ncbi.nlm.nih.gov/cdd) programs. These sequences were compared with Arabidopsis and tomato CAMTA proteins using ClustalW2 program (http://www.ebi.ac.uk/Tools/msa/clustalw2/) with default settings and were viewed by GeneDoc. Those containing a CG-1 domain, an ANK repeat domain and a CaM binding (CaMB) domain were recognized as CAMTA proteins. CAMTAs in a given species were named in accordance with sequence similarity to Arabidopsis CAMTAs.

### Phylogenetic, gene structure, and CaMB domain analyses of *CAMTA* genes

Multiple sequence alignments of the full-length CAMTA proteins from representative plant species were conducted using clustalX 2.01 program (Larkin et al., 2007). The phylogenetic tree was constructed using MEGA 5.0 (Tamura et al., 2011) with maximum likelihood (ML) method and a bootstrap test was performed with 1000 replicates. The gene structure was analyzed with the online Gene Structure Display Server (GSDS, http://gsds.cbi.pku.edu.cn/index.php) (Guo et al., 2007). The sequence logos of CaMB domain were generated using the Geneious software (v6.1.6) package (http://www.geneious.com/).

### Protein interaction network analysis

The online database resource Search Tool for the Retrieval of Interacting Genes/Proteins (STRING) version 10 (http://string-db.org/newstring_cgi/show_input_page.pl?) was used for prediction of protein interaction network. The prediction was performed with database default settings apart from the score-cutoffs which were re-adjusted to the value of 0.5. The STRING database integrates information from diverse data such as experimentally and manually curated protein-protein interactions, gene neighborhood, gene fusion, gene co-occurrence, as well as gene co-expression and text-mining is applied to uncover statistical and/or semantic links between proteins (Szklarczyk et al., 2015).

### Plant material and inoculation analysis

Arabidopsis plants of Col-0 and six *CAMTA* mutants (*Atcamta1-6*) were grown in a growth chamber at 21–22°C under a 15-h light/9-h dark photoperiod. For pathogen inoculation, the bacterial pathogen *Xanthomonas oryzae* pv. *oryzae* (*Xoo*) PXO99^A^ were incubated overnight at 28°C on NA liquid medium containing carbenicillin (50 μg/ml). The bacterial cells were collected by centrifugation and then diluted into suspensions to a concentration of OD_600_ 1.5 using sterilized ddH_2_O. Leaves of 4 week-old Arabidopsis plants were used for *Xoo* inoculation by needleless syringe infiltration. After infiltration, plants were grown at 27°C under a 16 h-light/8 h-dark photoperiod.

### Detection of ROS

*Xoo* inoculated leaves of *Atcamta* mutant and wild-type Col-0 plants were detached and stained with 3,3-diamino benzidine hydrochloride (DAB) (1 mg/mL) as previously described (Li et al., 2015). The H_2_O_2_ elicited by the PAMP peptide flg22 (100 nM) in leaf discs of *Atcamta3* mutant and wild-type Col-0 plants were measured using a Microplate Luminometer (TITERTEK BERTHOLD, Germany) following previously described protocol (Saand et al., 2015). For each experiment, 10 leaves were collected for each genotype. All experiments were conducted three times independently. The quantitative measurement data were statistically analyzed using SPSS software (Version 19.0, IBM, USA) and represent means ± standard error.

### Gene expression analyses by real-time PCR

Total RNA was isolated by Trizol (TAKARA, Japan) extraction according to the manufacturer's instructions. RNA was treated with DNase I (TAKARA, Japan) and reverse-transcribed into cDNA using the PrimeScript RT reagent kit (TAKARA, Japan). The obtained cDNAs were used for gene expression detection analysis with real time quantitative PCR. qRT-PCR was conducted in StepOne Real-Time PCR System (Applied Biosystems, USA) using SYBER Premix Ex Taq reagents (TAKARA, Japan) following the program: 95°C for 30 s, 95°C for 5 s, and 60°C for 45 s for 40 cycles. To normalize the sample variance, Arabidopsis *ACTIN8* gene served as an internal control. Relative gene expression values were calculated using the 2^−ΔΔCt^ method. The primers used for qRT-PCR analysis are listed at Table S5. For the statistical analysis of the gene expression data, ANOVA (analysis of variance) analysis was performed with SPSS software (Version 19.0, IBM, USA). Significance of the differences between mean values was determined with Duncan's multiple range test (DMRT) or Student's *t*-test.

## Results

### Identification of CAMTA family in 35 plant species

**Figure 1:**
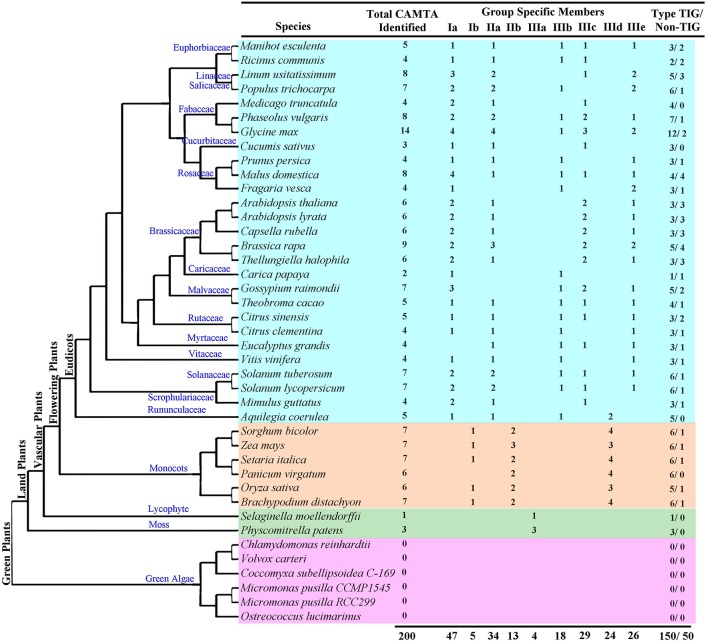
**CAMTAs identified in this study**. Phylogenetic tree for the plant species investigated in this study was shown.

Based on domain composition analyses of sequences retrieved from BLAST search using Arabidopsis CAMTAs as query, 200 CAMTA sequences were identified from 35 plant species, including one moss, one lycophyte, six monocots, and 27 eudicots (Figure 1 and Table S1). They were named in accordance with their phylogenetic relationship with the six Arabidopsis CAMTAs (Table S1). All these CAMTA proteins possessed a CG-1 domain, an ANK repeat domain, an IQ domain and a CaM binding (CaMB) domain in turn from the N terminus to the C terminus. One rice gene (Os03g27080), which was previously identified as a CAMTA (Choi et al., 2005), lacked CG-1 domain and thus was excluded from this analysis. In order to understand the evolutionary origin of the *CAMTA* genes, we also performed searches for *CAMTA* genes in the currently released 6 chlorophyta genomes. No gene containing both a CG-1 domain and an IQ domain or a CaMB domain was identified in these algal genomes (Figure 1). However, CAMTA-like proteins that carried a CG-1 domain, a TIG domain and an ANK repeat domain but lacking IQ or CaMB domains were found in *Ostreococcus lucimarinus* (Protein ID 26252) and *Coccomyxa subellipsoidea* (Protein ID 61775) (Figure S1). These results demonstrate that the CAMTA family is universally present in multicellular land plants but absent in unicellular eukaryotes and *CAMTA* genes may have evolved before the transition from non-vascular to vascular land plants.

The size of CAMTA family differed in the moss, lycophyte and higher flowering plant species. The moss *Physcomitrella patens* and the lycophyte *Selaginella moellendorffii* contained only three and one CAMTA(s), respectively, while higher flowering plant species generally carried 4–8 CAMTAs except soybean and *Brassica rapa*, which possessed 14 and 9, respectively, and papaya and cucumber, which surprisingly bore only two and three, respectively (Figure 1 and Table S1).

### Phylogeny of CAMTA proteins in plant

**Figure 2:**
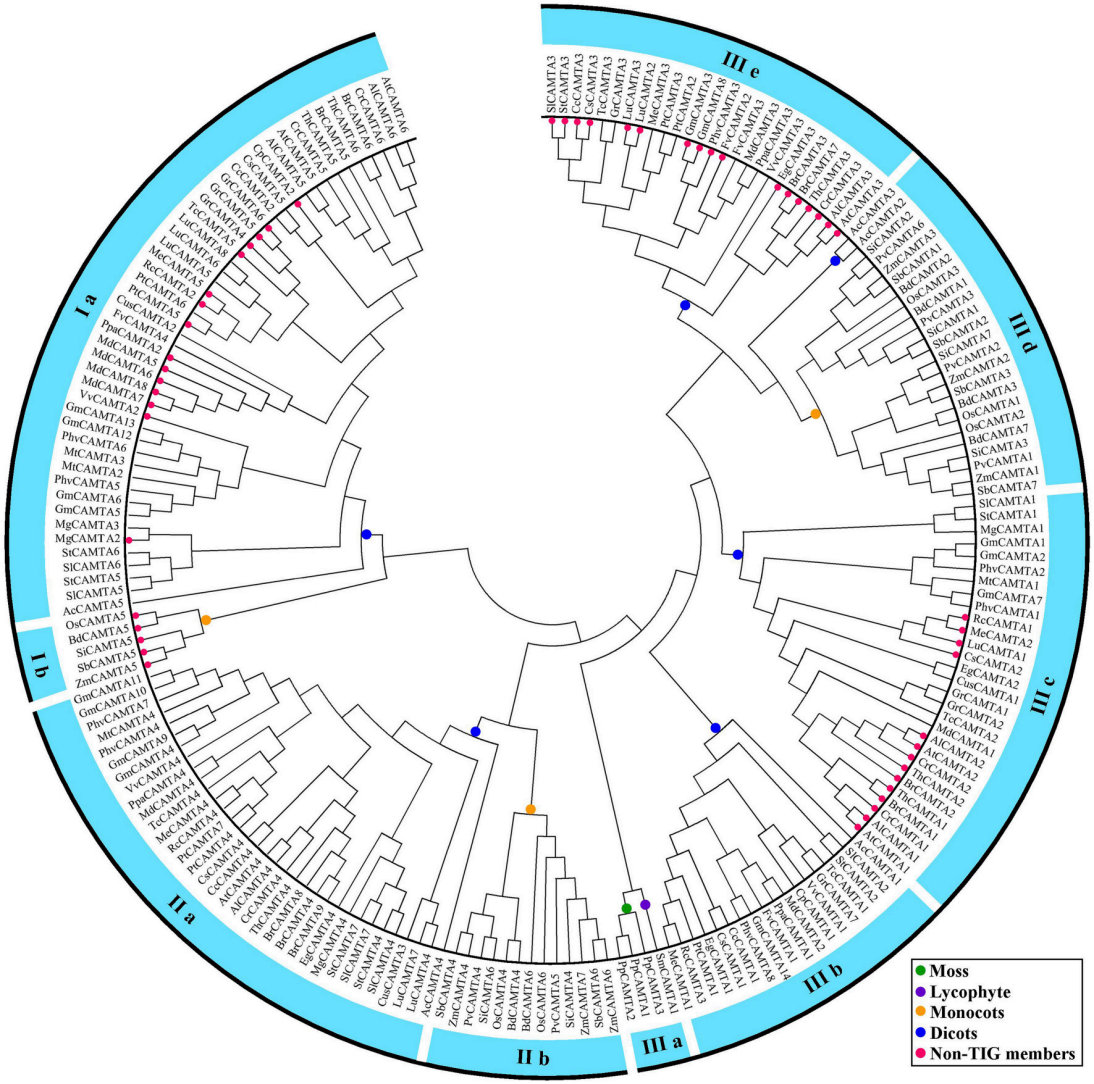
**Phylogenetic tree of 200 plant CAMTA proteins identified in this study**. The tree was constructed using Clustalx program by maximum liklihood (ML) method with bootstrap of 1000 in MEGA5. The colored dots symbolize the species to which the proteins in each clade belong.

To investigate the evolution of plant CAMTA proteins, we constructed a maximum likelihood (ML) tree based on the alignment of full-length CAMTA proteins (Figure 2). The phylogenetic tree showed that the 200 CAMTA proteins from 35 plant species clustered into three major groups (I–III) with groups I and II further divided into two subgroups while groups III separated into five subgroups with robust bootstrap support (Figure 2, Figure S2). Intriguingly, all four CAMTAs from the lower non-flowering land plants (moss and lycophyte) gathered into the group IIIa, while the remaining 196 CMATAs from the higher flowering land plants formed the other four subgroups of the group III as well as other two groups (I and II), demonstrating that the CAMTA family originated before the divergence of the non-flowering and flowering land plants. Both monocots and eudicots contained CAMTAs of each major group (I–III) but different subgroups. CAMTAs of monocots were distributed in group Ib, IIb, and IIId, while those of dicots congregated in group Ia, IIa, IIIa, IIIb, IIIc, and IIIe (Figures 1, 2 and Figure S3), revealing that *CAMTA* genes shared a common ancestor before the divergence between monocots and dicots and probably even before the divergence between higher flowering plants and lower non-flowering land plants. It is obvious that monocot and dicot CAMTAs underwent evolutionary diversification separately. Lineage-specific expansion and divergence events for CAMTAs obviously occurred in both monocots and dicots. For monocots, only one copy of non-TIG CAMTA appeared in group Ib, while TIG type CAMTAs occurred in group IIb and IIId, which underwent duplication once and twice, respectively. The evolution of CAMTAs was apparently similar for each monocot species. For dicots, group Ia CAMTAs contained both TIG and non-TIG types of CAMTAs depending on species, and the duplication frequency was also species-dependent. Group IIa CAMTAs were all TIG type and seldom underwent duplication. In group III, IIIb CAMTAs were all TIG type and no duplication occurred. IIIc CAMTAs containing both TIG and non-TIG types depending on plant species and duplication occurred only in some species, while IIIe CAMTAs were mostly non-TIG type and duplication occurred only in some species.

### Pattern of exon/intron structure of plant *CAMTA* genes

**Figure 3:**
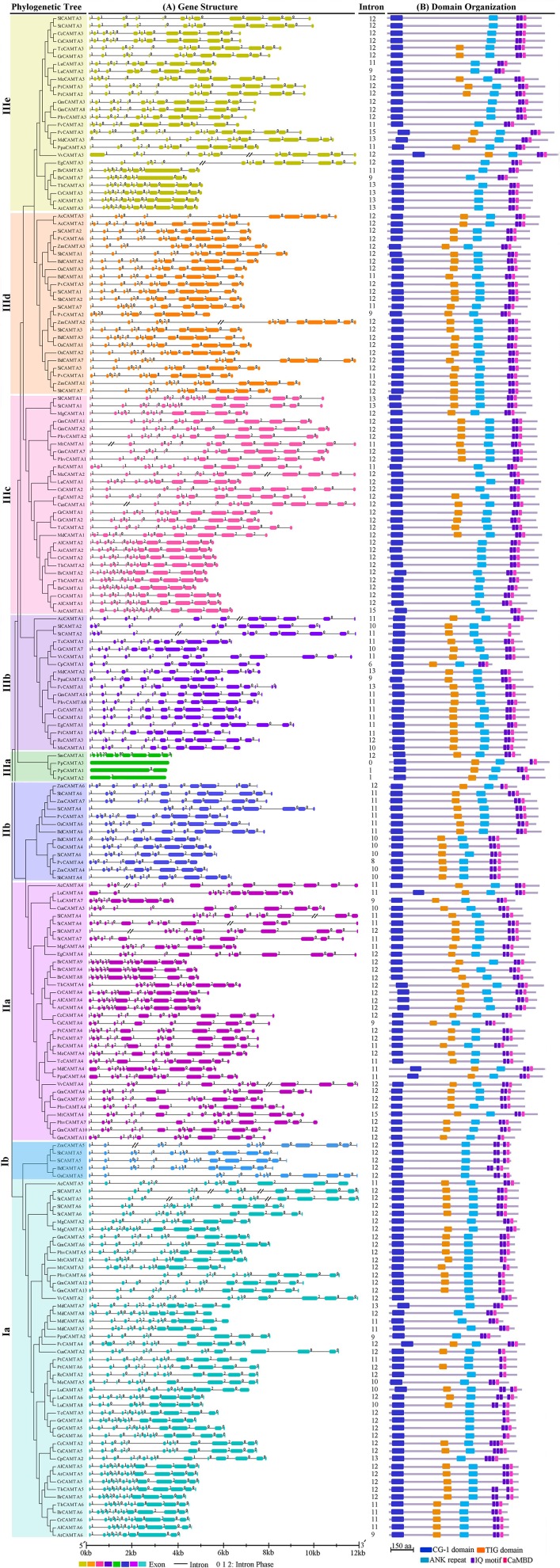
**Exon/intron structure and domain organization of the 200 CAMTAs identified from 35 plant species. (A)** Exon/intron gene structure. The untranslated region (UTR) sequences are not shown. The intron phases are indicated as numbers 0, 1, and 2. The exons and introns are drawn to scale except for AcCAMTA1, AcCAMTA4, CusCAMTA1, EgCAMTA3, MeCAMTA2, MtCAMTA1, SlCAMTA4, SlCAMTA5, SlCAMTA7, StCAMTA2, StCAMTA4, StCAMTA5, VvCAMTA3, VvCAMTA4, ZmCAMTA2, and ZmCAMTA5 for which long introns are denoted by a “//.” **(B)** Schematic diagram showing the domain organization of CAMTA proteins. The domains and motifs are drawn to scale.

The exon/intron structure pattern of the 200 *CAMTA* genes identified from 35 plant species was comparatively analyzed. Generally, the gene structure, including number, size, phase, and insertion sites of introns, was similar among the CAMTA orthologs in different species of flowering plants but was dramatically different in paralogs of a given species. Collectively, 59.0 and 21.5% of the identified *CAMTA* genes contained 12 and 11 introns, respectively, whereas only 7.0 and 12.5% of the genes carried introns of over 12 and less than 11, respectively. Nevertheless, intron number of *CAMTA* genes varied in plant lineages, which was 0–1 in moss, 12 in lycophyte, while dominantly 12 and 11 with a range from 6 to 15 in flowering plant species (Figure 3A and Table S2). Intron phase indicates the position of introns relative to the codon. Introns of phase 0, 1, and 2 are located between codons, between the first and second nucleotides of a codon, or between the second and third nucleotides of a codon, respectively. The results showed that the intron phase pattern of the unique *CAMTA* gene in the lycophyte (110201000200) was very similar to the dominant intron phase patterns of the 12-intron *CAMTA* genes of the flowering plant species (110201100200, 110201200100). Besides the intron phase pattern, the intron insertion sites of the 12-intron *CAMTA gene*s in the lycophyte and flowering plant species were very similar as well. These data provide strong support that they had a common origin and a 12 introns configuration might be an ancestral structure for *CAMTA* genes in vascular plant species. Additionally, the diverse number (6–15) of introns of *CAMTA* genes in vascular plants indicated that a significant number of intron loss and gain events occurred during the structural evolution of the *CAMTA* gene family. Nevertheless, although the *CAMTA* genes contained diverse number of introns, the insertion sites of both terminal introns, which included the first five and last four, of various *CAMTA* genes were highly conserved. In majority of *CAMTA* genes, insertion of the first five introns resulted in very small first five exons while insertion of last four introns caused three large exons located at position two to four from 3 prime and a very small exon at the end of 3 prime. The different number of introns from various *CAMTA* genes only led to distinct insertion in the middle part of the genes, which was conserved in genes of the same evolutionary groups or subgroups containing the identical number of introns (Figure 3A).

The gene structure was significantly different between *CAMTA* genes of different groups or even between those within the same groups mostly for members of the same species. Groups Ia, IIa, IIIb, IIIc, and IIIe consisted of dicot CAMTAs. Among them, group IIIb was dominated by 11-intron genes with a phase profile of 11020100200, while groups Ia, IIa, IIIc, and IIIe were all dominated by the 12-intron genes although they also contained some other genes, especially 11-intron genes. Nevertheless, the intron phase profile and/or intron size varied in the genes of these groups. Group Ia, IIa, and IIIe 12-intron *CAMTA* genes exhibited mainly a phase profile of 110201100200, while group IIIc 12-intron *CAMTA* genes displayed diverse phase profiles depending on plant species. *CAMTA* genes of woody plant species such as *Eucalyptus grandis, Citrus sinensis, Theobroma cacao*, and *Gossypium raimondii*, had a phase profile of 110201100100, while those of leguminous and cruciferous plants possessed two types of phase profiles, one was identical to the dominant one (110201100200) while the other was 112201100200 for legumes and 1102011(2/0)0100 for crucifers. Unlike the above-mentioned groups, groups Ib, IIb, and IIId constituted monocot CAMTAs. *CAMTA* genes of group Ib identically contained 12 introns with a phase profile of 11020100200, those of group IIId was also dominated by 12 introns but with a phase profile of 110201x00200, while those of groups IIb carried 11 or 10 introns with a phase profile of 11020100200 and 1102010200, respectively (Figure 3A). These data demonstrated that *CAMTA* genes of monocots and dicots underwent evolution in parallel.

In addition, it was obvious that the majority of plant species contained *CAMTA* genes with various numbers of introns (Table S3). The intron composition of *CAMTA* genes was distinct in different plant species. For example, papaya contained two *CAMTA* genes of 6 and 13 introns, respectively. Strawberry possessed four genes of 11, 12, 13, and 15 introns, respectively. Peach carried four genes of 9, 9, 11, and 11 introns, respectively. Arabidopsis bore six genes of 9, 12, 12, 12, 13, and 15 introns, respectively. However, two legumes, soybean and bean contained the highest number of *CAMTA* genes (14 and 8), all of them possessing 12 introns except one, which had 11 (Table S3). These results indicated that a significant number of intron loss and gain events occurred in many but not all plant species during the structural evolution of the *CAMTA* gene family. In addition, among the six monocots, five contained one gene of 10 introns, one or two genes of 11 introns and 4–5 genes of 12 introns. The exception was *Panicum virgatum*, which had six genes of 8, 9, 11, 11, 12, and 12 introns, respectively (Table S3). This implied that the gene structural evolution in monocots was very similar.

### Domain and motif composition of plant CAMTAs

According to the database searching results using NCBI-CDD and Pfam programs, all 200 CAMTA proteins, including those from moss and lycophyte, contained a CG-1 domain, an ANK repeat domain and a CaMB domain. All CAMTAs but two from tomato and potato possessed an IQ domain as well (Figure 3B). However, whether there was a TIG domain and how many motifs of IQ existed in the IQ domain differed in plant CAMTAs. Among the 200 CAMTAs, 50 did not contain a database-recognizable TIG domain. They were widely distributed in flowering plant species. These proteins were called non-TIG CAMTAs hereafter. All the three CAMTAs in moss and the unique one in lycophyte carried a TIG domain. However, all higher flowering plant species under this study contained multiple TIG class CAMTAs as well as 1–4 non-TIG class CAMTAs except four species *Panicum virgatum, Aquilegia coerulea, Cucumis sativus*, and *Medicago truncatula*, which only contained TIG class CAMTAs (Figures 2, 3B). This result reveals that non-TIG class CAMTAs are newly evolved in flowering land plants after divergence from the non-flowering plants. Moreover, existence of the non-TIG class of CAMTAs in higher flowering plants was group/subgroup-dependent. They clustered solely in group I and subgroups IIIc and IIIe (Figures 2, 3B). Remarkably, the non-TIG CAMTAs of monocots uniquely gathered in subgroup Ib. However, presence of those of dicots was species dependent. The non-TIG CAMTAs in solanaceous and leguminous species appeared only in subgroup IIIe; those from cruciferous and two *Citrus* species gathered in subgroups IIIc and IIIe; those in cotton, coco, castor, and cassava existed in subgroups Ia and IIIc, while those from peach, apple, and grape congregated in subgroups Ia and IIIe (Figures 2, 3B). The number of non-TIG CAMTAs varied in plant species. Most of plant species contained 1–3 non-TIG type CAMTAs. Some species carried an equal number of non-TIG and TIG CAMTAs. These species included four crucifers *Arabidopsis thaliana, A. lyrata, Thellungiella halophile*, and *Capsella rubella*, and an apple and a castor species (Figure 1). These data demonstrate that generation of non-TIG class CAMTAs contributed significantly to the expansion of CAMTAs in higher flowering plants especially in crucifers. In addition, the TIG domain was present in CAMTAs from moss and lycophyte species (Figures 1–3) and even in two putative CAMTA-like sequences of algae (Figure S1). Alignment of all the 200 CAMTAs demonstrated that the region corresponding to the TIG domain of TIG class CAMTAs existed in a similar position in the non-TIG CAMTAs. These results indicate that the TIG domain emerged first in ancestors of land plants and somehow mutation of some key amino acids in the TIG domain rather than deletion of this domain occurred in the non-TIG CAMTAs during evolution.

Another variation in plant CAMTAs was the number of the IQ motifs existing in the IQ domain. Although the dominant number of the IQ motif was two, more or less IQ motifs did occur in some CAMTAs. For example, two CAMTAs from tomato and potato in subgroup IIIb did not contain any database-recognizable IQ motif. A total of 12 CAMTAs carried only one IQ motif. These included 10 CAMTAs in subgroup Ia, among which were five leguminous, two *Populus*, one cucumber, and one castor CAMTAs, one soybean CAMTA in subgroup IIa and one maize CAMTA in subgroup IIb. However, all five monocot CAMTAs in subgroup Ib and eight dicot CAMTAs in subgroup Ia, including three from flax, two from Citrus, one each from papaya, *Thellungiella halophila* and *Brassica rapa*, were composed of three IQ motifs. Moreover, in moss, one CAMTA had only one IQ motif, the remaining two CAMTAs consisted of two IQ motifs; while in the lycophyte, the unique CAMTA owned two IQ motifs. These data suggest that the ancestor CAMTA(s) might own two IQ motifs and the number of IQ motifs significantly affects the clustering of CAMTA proteins in the phylogenetic tree.

### Conservation of CaMB domain of plant CAMTA proteins

**Figure 4:**
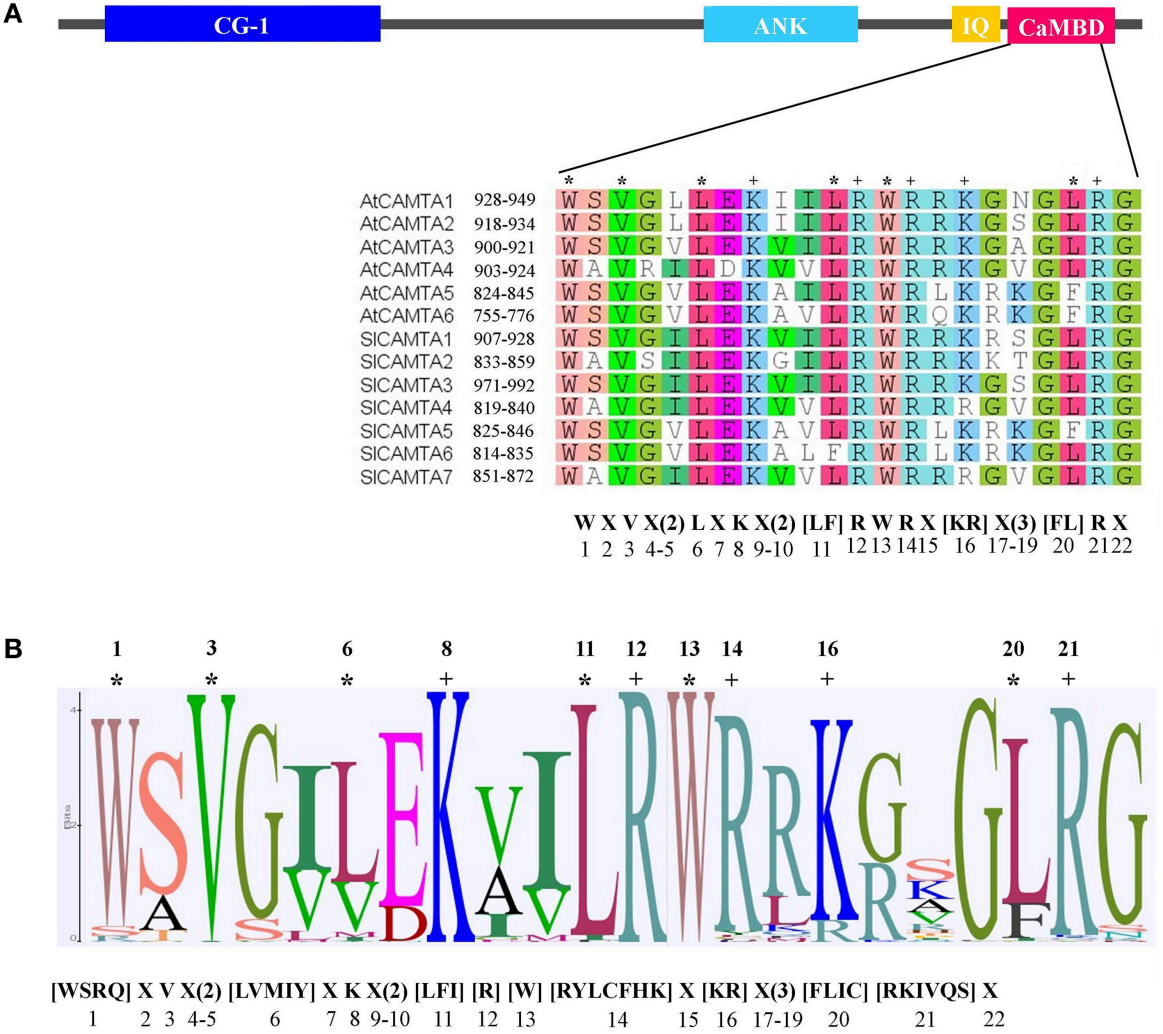
**The conserved motif in CaMB domain of plant CAMTAs. (A)** Functionally proved motif in Arabidopsis and tomato CAMTAs. Alignment of the CaMB domain in 6 Arabidopsis and 7 tomato CAMTAs is shown. The functional residues in CaMB domain of these CAMTAs are indicated in the motif below the alignment. In the square brackets “[]” are the amino acids allowed in this position of the motif; “X” represents any amino acid and the round brackets “()” indicate the number of amino acids. **(B)** Sequence logo of the CaMB domain of 200 CAMTA proteins identified in this study. A “^*^” and a “+” indicate a hydrophobic and a positively charged residue respectively. The corresponding conserved motif is shown below the logo.

CaMB domain is indispensable to CAMTAs. The functional residues in this domain required for CaM binding have been identified in Arabidopsis and tomato and thereby a functional motif (W X V X(2) L X K X(2) [LF] R W R X [KR] X(3) [FL] R X) for CaMB domain has been suggested (Bouché et al., 2002; Yang et al., 2012; Figure 4A). To clarify the conservation of this domain among plant CAMTAs, the domain sequences of the 200 CAMTAs identified in this study were aligned. The alignment revealed a conserved motif for functional residues as [WSRQ] X [VI] X(2) [LVMIY] X K X(2) [LFI] R W [RYLCFHK] X [KR] X(3) [LFIC] [RKIVQS] X (Figure 4B). In this motif, the first residue is the hydrophobic W in 186 of the 200 sequences. However, this W was replaced by S, R, or Q in the 13 monocot CAMTAs in subgroup IIb and also by R in one dicot sequence (EgCAMTA1) of subgroup IIIb. The third residue was identically V except one sequence (PtCAMTA6) of group Ia, in which it was substituted by a similar residue I. The sixth residue was dominated by L or V except 11 CAMTAs of subgroups IIa, IIIb, IIIc, and IIIe, where the corresponding residue was M, I, or Y. The 8th, 12th, and 13th residues were consistently K, R, and W, respectively, in all 200 CAMTAs. The 11th residue was overwhelmingly L except six sequences of subgroups Ia and IIb, in which this residue was I or F. The 14th residue was dominated by R except 10 sequences of subgroups Ia, IIa, and IIb, in which this residue was Y, L, F, H, C, or K. The 16th residue was K or R. The 20th residue was mostly L except CAMTAs of subgroup Ia, in which it was F or L. The 21th residue was dominated by R except six sequences of subgroups Ia, IIa, and IIIb, in which this residue was I, K, S, Q, or V. Generally, this motif was highly conserved in CAMTAs of monocot and dicot flowering plants as well as moss and lycophyte non-flowering plants. The only significant variations were the first and 20th residues. The first residue was dominantly W but was S, R, or Q in the 13 monocot CAMTAs of subgroup IIb, while the 20th residue was overwhelmingly L in CAMTAs of all subgroups except Ia, in which it was dominated by F, followed by L (Figure 4, Figure S4). Additionally, comparison of this motif with the experimentally confirmed one for only Arabidopsis and tomato CAMTAs demonstrated that there was minor variation in 7 of the total 11 conserved amino acids at the positions of 1, 3, 6, 11, 14, 20, and 21 in the motif (Figure 4, Figure S4). Whether these small changes result in functional alteration remains to be experimentally validated. Collectively, these data demonstrated that with very few exceptions, the motif of CaMB domain was highly conserved in all land plant species.

### Protein interaction network of arabidopsis CAMTA family

**Figure 5:**
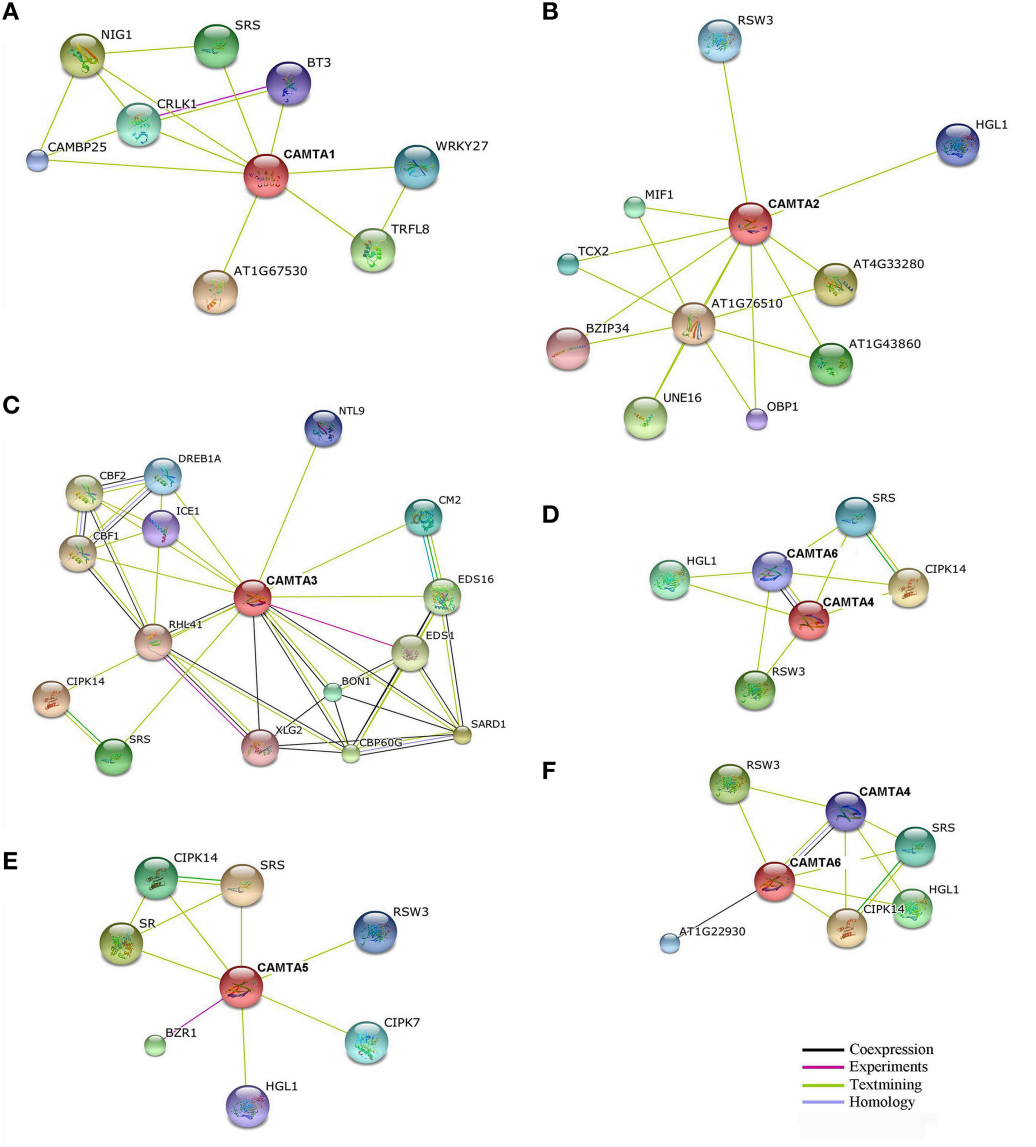
**Protein interaction network of Arabidopsis CAMTAs**. The potential interactors of AtCAMTA1 **(A)**, AtCAMTA2 **(B)**, AtCAMTA3 **(C)**, AtCAMTA4 **(D)**, AtCAMTA5 **(E)**, and AtCAMTA6 **(F)** were predicted using STRING program. The various ways of interactions are illustrated with different colored connective lines.

A total of 38 unique proteins were predicted as potential interactors of the six Arabidopsis CAMTAs (AtCAMTA1-6) using the STRING program when the confidence value was set as 0.5 (Figure 5 and Table S4). Among them, 8, 10, 15, 5, 7, and 6 proteins were identified as possible interactors of CAMTA1, 2, 3, 4, 5, and 6, respectively, and some of them obviously interacted with more than one AtCAMTAs (Figure 5 and Table S4). Remarkably, among the 38 *AtCAMTA* associated proteins, 23 (60.5%) were DNA binding transcription factors, including SARD1, CBP60G, BZR1, NIG1, TRFL8, ARID/BRIGHT, DREB1A/CBF3, NTL9, ICE1, WRKY27, AP2/B3-like, UNE16, MIF1, TCX2, RHL41, CBF1, BT3, OBP1, CBF2, bZIP34 (Table S4). This result suggests that transcriptional regulation of the target genes might be the dominant mechanism of the AtCAMTA-associated functional regulation. The additional partners of AtCAMTAs were predicted to be three Ca^2+^/CaM-regulated protein kinases CIPK7, CIPK14, and CRLK1, two Ca^2+^-dependent phospholipid signaling proteins BON1 and EDS1, a CaM-binding protein CAMBP25, a SA biosynthesis key enzyme EDS16, a G protein XLG2, a ubiquitin-protein ligase, two serine metabolism enzymes SRS and SR, and two glucosidases RSW3 and HGL1 (Table S4). These data suggest that AtCAMTAs act together with other Ca^2+^ signaling components to regulate Ca^2+^-related biological processes. Moreover, *AtCAMTA4* and *AtCAMTA6* were mutually predicted to interact with each other, indicating that these two AtCAMTAs either directly interact or function similarly in the same pathways.

In addition, different AtCAMTAs had redundant but not identical predicted interactors. *AtCAMTA3* possessed 15 interactors, including SARD1, CBP60G, EDS1, and EDS16, which are known to be involved in plant disease resistance (Zhang et al., 2010; Wang et al., 2011). Among them, *EDS1* is an experimentally confirmed target of *CAMTA3* (Du et al., 2009; Nie et al., 2012), supporting the reliability of the observed interaction network. The other interactors are mostly involved in abiotic stress tolerance. For example, it has been reported that the activation of *CBF2* by *CAMTA3* affects plant response to cold and freezing tolerance (Doherty et al., 2009). Taken together, these data indicated that *AtCAMTA3* is involved in both plant defense and abiotic stress responses. Potential interactors of the other five AtCAMTAs are mainly involved in plant response to environmental and abiotic stress stimuli, indicating that these AtCAMTAs play a role in these responses.

**Figure 6:**
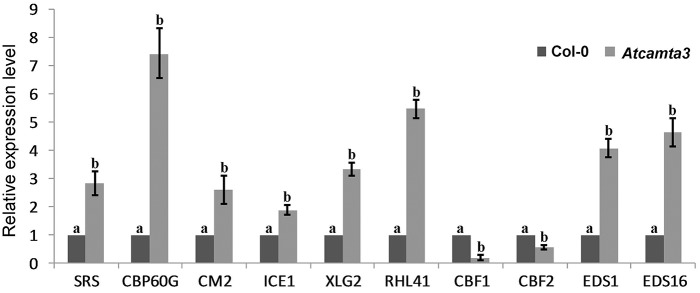
**Expression profiles of predicted AtCAMTA3-targeted genes in Arabidopsis**. Expression of 10 predicted AtCAMTA3-targeted genes *SRS, CBP60G, CM2, ICE1, XLG2, RLH41, CBF1, CBF2, EDS1*, and *EDS16* in *Atcamta3* mutant and Col-0 control plants were analyzed by qRT-PCR with gene-specific primers listed in Table S5. Significant difference between expression values of the target genes and that of control is indicated as lowercase letters (*p* < 0.05, DMRT). Data represent the mean ± SE of three independent experiments.

CAMTAs regulate expression of target genes by directly binding with the CGCG *cis*-element of their promoters (Yang and Poovaiah, 2002; Du et al., 2009). To further understand the functional mechanism of AtCAMTAs, all the 38 predicted interactors were examined for presence of the CGCG *cis*-element in their promoters. Prediction analysis showed that 16 interactors contained at least one CGCG element in their 1.5 kb sequences upstream of the start codon. These included transcription factor genes *CBP60G, BZR1, ICE1, RHL41, CBF1, BT3*, and *CBF2*, and other genes *CM2, CRLK1, EDS1, CAMBP25, EDS16, XLG2, SRS*, and *RSW3* (Table S4). This result indicated that AtCAMTAs might target these genes to regulate biological processes. Intriguingly, *AtCAMTA6* carried a CGCG element in its upstream sequence (Table S4). This implied that *AtCAMTA4* might regulate the expression of *AtCAMTA6*, indicating the direct interaction between *CAMTA* members. To provide further evidence about whether these CGCG *cis*-element-containing genes are potential targets of AtCAMTAs, we analyzed the expression of 10 predicted *AtCAMTA3* target genes containing a CGCG element in wild-type and mutant plants. The checked genes included *SRS, CBP60G, CM2, ICE1, XLG2, RHL41/ZAT12, CBF1, CBF2, EDS1*, and *EDS16/ICS1*. As shown in Figure 6, expression of all checked genes significantly altered in *camta3* mutant plants compared with that in wild-type plants. However, the alteration of expression differed among the genes. Expression of 8 out of 10 checked genes significantly increased by up to 7.5-fold for *CBP60G*, while that of the remaining two genes, *CBF1* and *CBF2* are reduced in *camta3* mutant plants compared with that in wild-type plants (Figure 6). These results indicate that *AtCAMTA3* may negatively regulate the expression of the eight target genes, while positively regulating the expression of two *CBF* genes, and suggest that these genes are likely to be the target genes of AtCAMTA3.

### Arabidopsis *CAMTA3* negatively regulated nonhost resistance to bacterial pathogen *Xoo*

**Figure 7:**
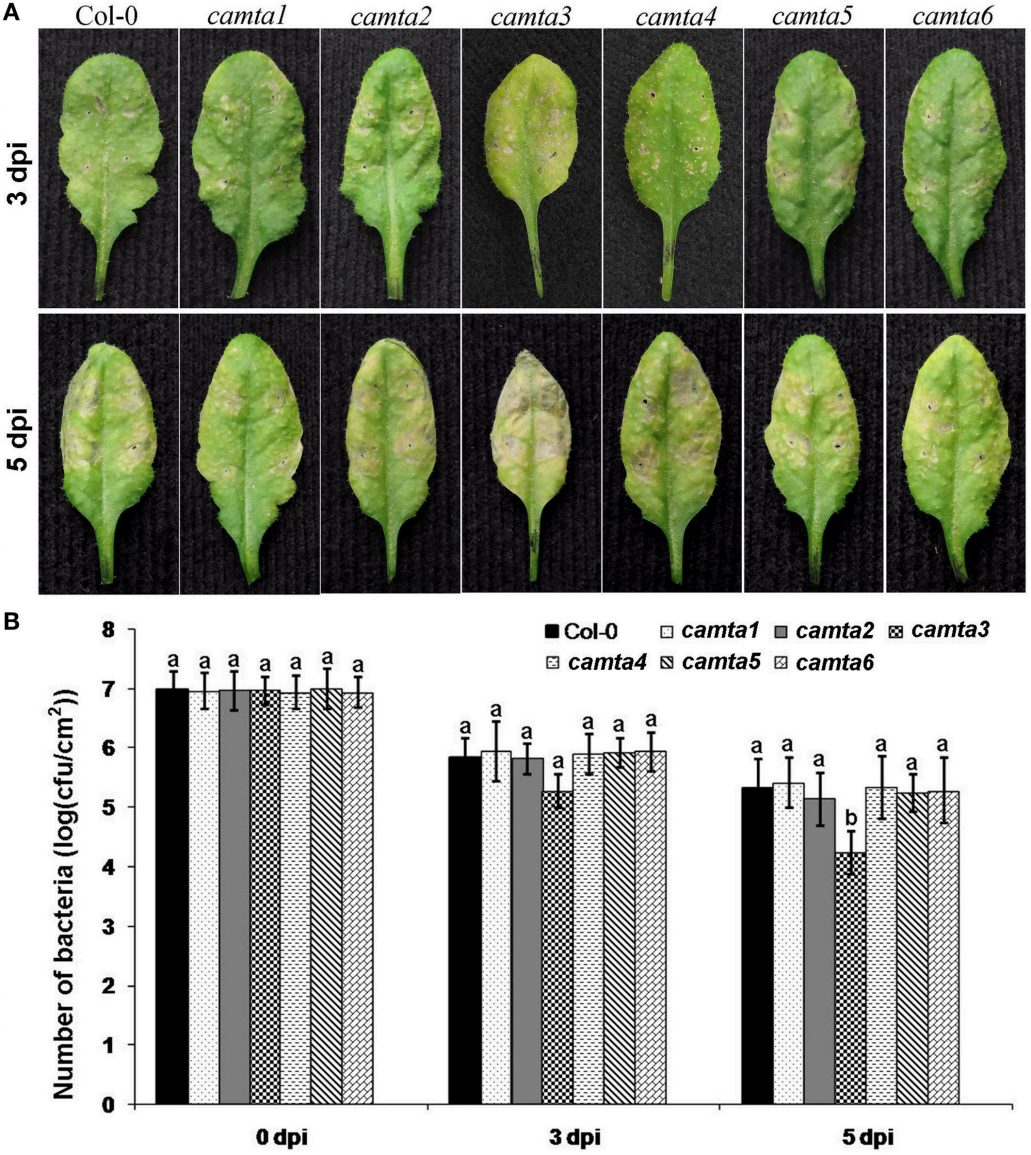
*****Atcamta3*** mutant plants exhibited enhanced HR and nonhost resistance to *Xoo*. (A)** Hypersensitive response symptoms of *Atcamta* mutant and Col-0 wild-type plant leaves inoculated with *Xoo* (OD_600_ 1.5). Photographs were taken at 3 and 5 dpi. **(B)** *Xoo* bacterial numbers counted from inoculated leaf areas at 0, 3, and 5 dpi. At least five plants were examined for each experiment and the experiments were conducted three times independently. The data in all statistical analyses represent the mean ± SE of three experiments. Significant difference is indicated as small letters (*p* < 0.05, DMRT).

To understand the functions of AtCAMTAs in nonhost resistance, plants of the six Arabidopsis CAMTA mutants (*Atcamta1-6*) were inoculated with the nonhost bacterial pathogen *Xoo*. *Atcamta3* leaves started to exhibit hypersensitive necrosis at 3 dpi, and turned completely necrotic at 5 dpi, while wild-type and other *Atcamta* plants did not display hypersensitive necrosis until 5 dpi (Figure 7). Furthermore, bacterial numbers in these leaves of various mutants was counted. Results showed that bacterial numbers of *Xoo* in *Atcamta3* leaves reduced dramatically at 5 dpi compared with in wild-type and other *Atcamta* leaves (Figure 7). These data revealed that *AtCAMTA3* negatively regulates HR and nonhost resistance to bacterial pathogen *Xoo* in Arabidopsis.

### Arabidopsis *CAMTA3* negatively regulated both the *Xoo*-induced and PAMP-elicited ROS accumulation

**Figure 8:**
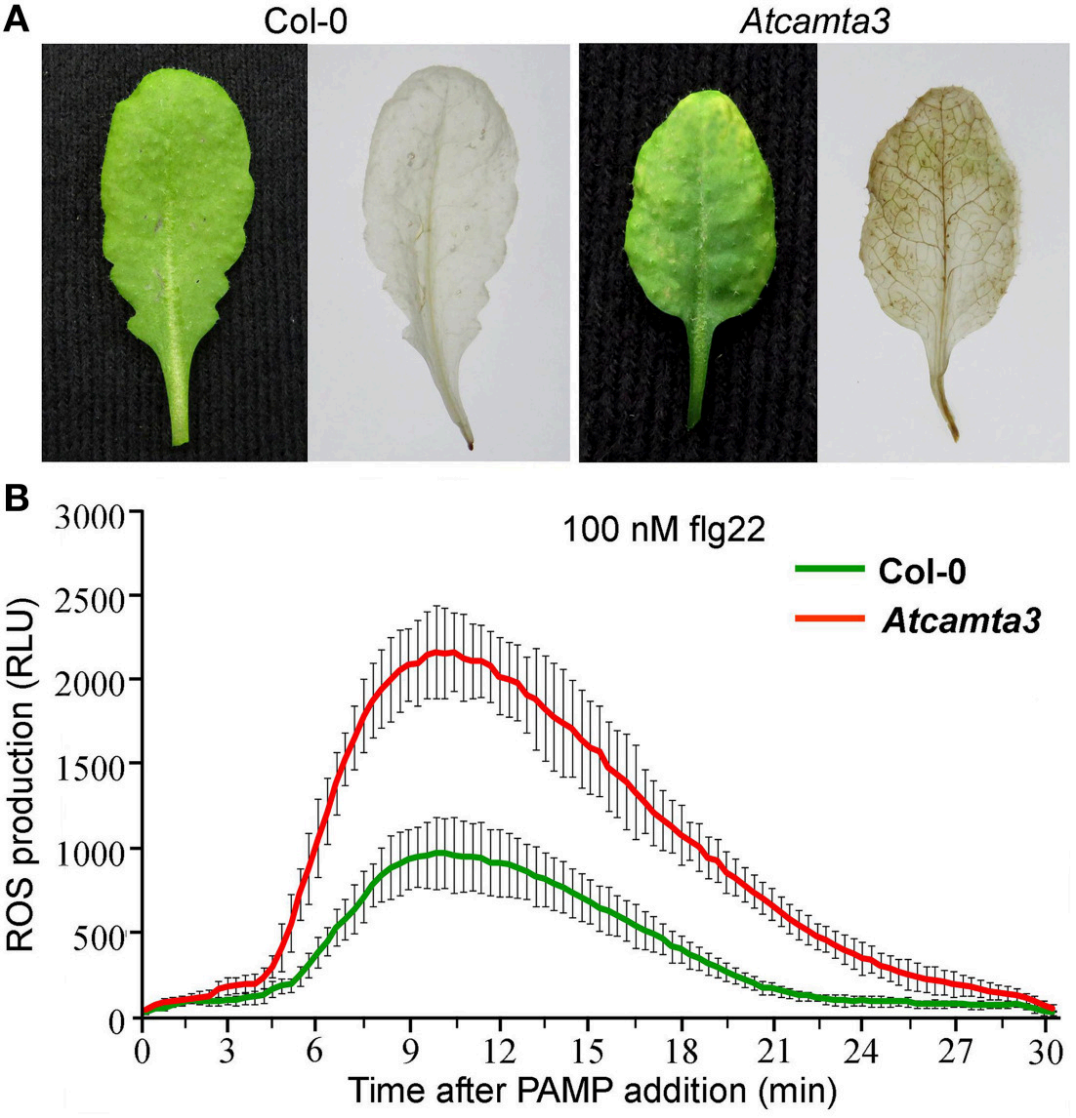
*****Atcamta3*** mutant plants accumulated higher level of ***Xoo***-induced and PAMP-elicited H_**2**_O_**2**_. (A)** Detection of H_2_O_2_ by 3,3-diaminobenzidine (DAB) staining analyses. The leaves of Col-0 and *Atcamta3* mutant plants at 24 h post-inoculated with *Xoo* were collected for DAB staining analyses. The same leaves before and after staining analyses were shown. **(B)** Dynamics of H_2_O_2_accumulation in response to flg22 elicitation in leaves of Col-0 and *Atcamta3* mutant plants. Flg22-triggered H_2_O_2_ bursts were measured using luminol-based assay in leaf discs of Col-0 and *Atcamta3* mutant plants. Data are shown as relative luminal units (RLU) and represent the mean ± SE of three independent experiments.

To probe the role of *AtCAMTA3* in H_2_O_2_ accumulation, the key factor of the nonhost resistance to *Xoo* (Li et al., 2015), leaves of *Atcamta3* mutant and wild-type plants were stained *in situ* with 3,3-diamino benzidine hydrochloride (DAB) at 24 h after *Xoo* inoculation. The leaves of *Atcamta3* mutant plants were stained brown, while those of the wild-type plants were not significantly stained at this time point (Figure 8A), demonstrating that *AtCAMTA3* negatively regulates the accumulation of *Xoo*-induced H_2_O_2_.

To further examine the role of *AtCAMTA3* in PAMP-elicited H_2_O_2_ accumulation, the H_2_O_2_ was monitored after supply with a bacterial PAMP flg22 (100 nM) to leaf disks of the *Atcamta3* mutant and wild-type plants. In *Atcamta3* mutant, H_2_O_2_ increased rapidly and peaked at 12 min post PAMP application to 2161 RLU, whereas the wild-type plants, although displaying similar dynamics of H_2_O_2_ accumulation showed a much lower peak value (971 RLU) (Figure 8B). This result indicates that *AtCAMTA3* negatively regulates the accumulation of flg22-elicited H_2_O_2_.

Collectively, these results reveal that Arabidopsis *CAMTA3* negatively regulated both the *Xoo*-induced and PAMP-elicited ROS accumulation and thereby affects the nonhost resistance to *Xoo* and flg22-trigged immunity.

### Arabidopsis *CAMTA3* negatively regulated expression of *CBP60G, EDS1*, and *NDR1* and was positively responsive to *Xoo* inoculation

**Figure 9:**
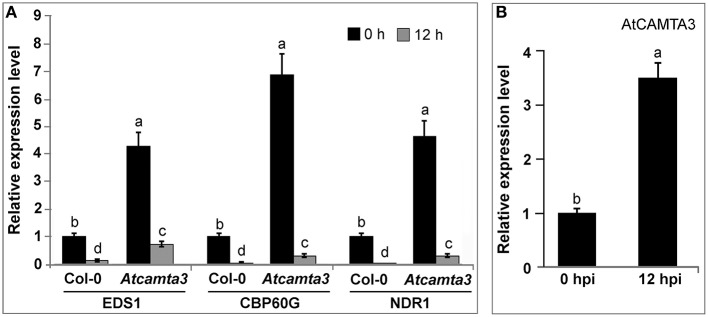
**Expression profiles of ***AtCAMTA3*** and its target genes ***EDS1***, ***CBP60G***, and ***NDR1*** in response to ***Xoo*** inoculation**. The expression levels of these AtCAMTA3-targeted genes in Col-0 wild-type and *Atcamta3* mutant plants were examined by qRT-PCR at 0 and 12 h post *Xoo* inoculation. **(A)** Expression dynamics of genes in response to *Xoo* inoculation in wild-type and *Atcamta3* mutant plants. **(B)** Expression of *AtCAMTA3* gene in response to *Xoo* inoculation in wild-type plants. Significant differences in gene expression are indicated by lowercase letters (*p* < 0.05, DMRT for **A** and Student's *t*-test for **B**). Data represent the mean ± SE of three independent experiments.

To further elucidate the mechanism of *AtCAMTA3* in regulating nonhost resistance to *Xoo*, we examined the expression of *EDS1, CBP60G*, and *NDR1*, three putative or confirmed target genes of *AtCAMTA3* playing important roles in plant disease resistance, in wild-type and *Atcamta3* plants before and after inoculating with *Xoo*. Compared with wild-type plants, expression of *EDS1, CBP60G* and *NDR1* in *Atcamta3* plants significantly was increased by 4.2-, 6.8-, and 4.6-folds, respectively (Figure 9A). Moreover, in wild-type plants, expression of *AtCAMTA3* was induced by 3.4-folds (Figure 9B), while expression of *EDS1, CBP60G*, and *NDR1* genes was dramatically reduced by 77.4, 96.3, and 96.8%, respectively, in response to *Xoo* inoculation at 12 hpi (Figure 9A). Together, these results showed that in wild-type plants, *AtCAMTA3* negatively regulated expression of *CBP60G, EDS1* and *NDR1* in response to *Xoo*, and implied that *AtCAMTA3* may negatively regulate nonhost resistance to *Xoo* via negatively regulating expression of *CBP60G, EDS1* and *NDR1*. Additionally, in *Atcamta3* plants, expression of *EDS1, CBP60G*, and *NDR1* genes was still significantly reduced in response to *Xoo* inoculation at 12 hpi (Figure 9A), indicating that factor(s) other than AtCAMTA3 might respond to *Xoo* inoculation to suppress the expression of these defense signaling genes in *Atcamta3* plants.

## Discussion

### Origin and evolution of *CAMTA* genes

There is no doubt that land plants originated from green algae and that most of the genes in the genomes of land plants were vertically inherited from their common ancestors (Lewis and McCourt, 2004; Wodniok et al., 2011). Our sequence similarity search against complete genome sequences of Viridiplantae species, including unicellular green algae, moss, lycophyte, and angiosperm suggested that *CAMTA* genes existed only in land plants (Figure 1). The earliest *CAMTA* genes we identified in this study were from the genome sequence of *Physcomitrella patens*, a model organism representing the bryophyta. This is similar to what has been suggested in the previous studies on animal species and yeasts that CAMTAs exist only in multicellular eukaryotes (Bouché et al., 2002; Finkler et al., 2007). However, although no CAMTA sequences were found in green algae, chlorophyta species contain CAMTA-like proteins with conserved CG-1, TIG, and ANK domains typical to those of embryophyta, while IQ/calmodulin-binding domain is encoded by a separate gene or associated with other proteins. In *Micromonas pusilla* CCMP1545, for example, a CAMTA-like and IQ/EF-hand binding site (according to analyses with Prosite and InterPro databases) encoding genes with NCBI accession number XM_003055634.1 and XM_003055633.1, respectively, are located on successive positions in the genome. In addition, one CAMTA-like protein that carried only a CG-1 domain, a TIG domain and an ANK repeat domain, but neither IQ or CaMB domains, was also found in *Ostreococcus lucimarinus* (Protein ID 26252) and *Coccomyxa subellipsoidea* (Protein ID 61775) (Figure S1). Thus, the most conceivable evolutionary scenario of *CAMTA* genes is likely to be the fusion of two separate genes encoding the CAMTA-like protein and IQ/CaM binding motif containing proteins, respectively, in the embryophyta lineage ancestor.

Gene fusion is a well-known process in molecular evolution and it has been indicated to be useful in predicting functionally associated proteins, including interactions, throughout genomes (Enright and Ouzounis, 2001). However, from gene structure analyses in green algae CAMTA-like genes and plant CAMTAs, the timing of CAMTA progenitor gene fusion remains mysterious. In analyzed species, the green algae CAMTA-like genes from *Ostreococcus lucimarinus, Micromonas sp*. RCC299, *Micromonas pusilla* CCMP1545, *Coccomyxa subellipsoidea* C-169, are intron-free and contain one, three, and 27 introns, respectively, while one of moss *CAMTA* genes exhibit zero or one introns, and the number rapidly increase to twelve introns in the lycophyte (Figure 3). Therefore, it is not clear whether the last common ancestor of plant CAMTAs was intronless, there was intron death after the fusion with the intron-free gene of IQ/EF-hand motif containing protein, or the two genes fused before or after moss divergence.

It is evident that the duplication of the moss primitive intron-free CAMTA took place after divergence of the lycophyte, and the intron invasion possibly occurred in moss and lycophyte simultaneously followed by rapid expansion in the latter. The angiosperm *CAMTA* genes evolved from that of the lycophyte homolog which underwent repeated duplications in their respective hosting plant species with subsequent intron enlargement and turnover, and exon rearrangements and skipping as well. Structures of genes encoding group I and III CAMTAs revealed conservation of intron phases with few exceptions, while exon-intron configurations in group II showed a slight deviation (Figure 3). Contrary to groups I and III in which 12 intron configuration accounted for the majority of the genes irrespective of taxonomic groups, group II monocot and dicot *CAMTA* genes are structurally different with 11 and 12 introns dominating in monocots and dicots respectively (Figure 3). However, it is speculative that group II CAMTA ancestor gene contained 11 introns given the conservation of this arrangement in monocots, its presence in the eudicot ancestor *Aquilegia coerulea* and in at least one species of each family except Brassicaceae and Fabaceae. On the other hand, in addition to intron number and phase, groups I *CAMTA* genes have other common features with members of those of group III such as reduced size of exon 9 in both group I and subgroup IIIc. Therefore, it is conceivable that angiosperm group I and III CAMTAs evolved from a single *CAMTA* gene which was most likely distinct from the ancestor of group II homologs, suggesting a biphyletic origin of angiosperm CAMTAs from their last common ancestor. This is consistent with the report that conserved intron positions and phases were gained only once in evolution (Putnam et al., 2007). Moreover, some *CAMTA* genes from either group are located on the same chromosome such as in rice, tomato, and poplar, for example, whereas group II rarely share chromosomes with either group I or III excepting cases such as in *Glycine max* and *Phaseolus vulgaris* which contain relatively large number of *CAMTA* gene copies (Table S1).

The major event that followed the duplication of the lycophyte originated *CAMTA* gene in the early ancestor of all angiosperm, was probably the intron enlargements along with rearrangement of exons 6 and 8 (exon 7 not involved) by fusion and recombination for group II and groups I and III ancestors, respectively, that resulted in reduction of intron number to 11 in group II ancestor gene while the number of exons/introns in group I and III ancestor *CAMTA* gene remained unchanged. The gene rearrangement also brought about the decrease and increase in size of exon 6 and 8, respectively, in CAMTA ancestral gene of group I and III (Figure S5). Other *CAMTA* gene copies within each group apparently arose mainly from chromosomal duplications and/or lineage-specific whole genome duplication during evolution of different plant species as depicted in chromosome or scaffold distribution (Table S1). Despite the possible monophyletic origin of group I and III angiosperm CAMTAs and gene structure similarities, the two groups must have separated prior to the earliest diversification of angiosperms since group I clusters sequences from all species without significant bootstrap supported distinct subgroups like those in group I except the cluster of monocots (Figure 3).

Evolvement of *CAMTAs* in land plant lineages from two separated genes encoding a CAMTA-like protein lacking a IQ/CaM binding domain and an IQ/CaM binding motif containing proteins, respectively, in green algae, may reflect the adaptation of land plants to the environmental changes. Fusion of two separated genes into one facilitates regulation of function in a biological process. Emergence of this way of more effective regulation also indicates the essential role of CAMTAs in the plant adaptation to the environmental changes.

The present study reveals that all but two CAMTAs identified in 35 plant species contain both IQ and CaMB domains (Figure 3). The reason that these two CaM binding domains exist together in the same protein is unclear. Previous reports demonstrated that the IQ domain binds to CaM in a Ca^2+^-independent manner, while the CaMB domain interacts with CaM in a Ca^2+^-dependent way (Bouché et al., 2002; Yang and Poovaiah, 2002; Choi et al., 2005; Finkler et al., 2007; Du et al., 2009; Yang et al., 2012). Therefore, the presence of both domains enables CAMTAs to interact with CaM in the absence as well as presence of Ca^2+^, leading to inactivating or activating the downstream components in response to different concentrations of Ca^2+^. Alternatively, given that several CaMs and many CaM-like (CML) proteins exist in a plant species (Zhao et al., 2013), IQ and CaMB domains may bind to different CaMs, and thus activate distinct downstream target genes, thereby regulate different biological processes. Moreover, we found that the number of the IQ motifs existing in the IQ domain varies in plant CAMTAs, with two being dominant, but also three and one distributed among 26 CAMTAs (Figure 3). Whether the number of the IQ motifs existing in the IQ domain affects CaM binding capacity and whether the CAMTAs containing varying number of IQ motifs bind to distinct CaM or CML and/or activate distinguishable downstream target genes requires further experimental examination.

Additionally, about one fourth of plant CAMTAs do not contain a TIG domain (Figure 3). This non-TIG class of CAMTAs obviously newly evolved in flowering land plants after divergence from the non-flowering plants, indicating that non-TIG CAMTAs occurred during plant adaptation to the environmental changes. The TIG domain is involved in non-specific DNA binding. It may affect the CG-1 domain-dependent substrate selection and binding and thus influence the scope of downstream target genes to be activated, thereby fine tuning the regulation of the target biological processes.

### Network inference of CAMTA proteins

To explore the functional pathways and regulatory gene networks of plant *CAMTA* genes, 38 potential interactors of the six Arabidopsis CAMTA proteins were predicted by using STRING program (Figure 5 and Table S4). The most significant finding of this analysis is that the majority of the *AtCAMTA* associated proteins are DNA binding transcription factors and/or Ca^2+^/CaM-regulated proteins (Table S4). Our result revealed that transcriptional regulation of the target genes might be the dominant mechanism of the *AtCAMTA*-associated functional regulation, and AtCAMTAs act together with other Ca^2+^ signaling components to regulate Ca^2+^related biological processes. Furthermore, we found that 16 potential interactors contained at least one CGCG CAMTA-binding element in their 1.5 kb sequences upstream of the start codon (Table S4). This result indicates that about half of the predicted interactors might be target genes whose expression is regulated by AtCAMTAs. To further support this possibility, we analyzed effect of CAMTA3 on the expression of 10 predicted CGCG element-containing interactors. We found that expression of all these genes changed significantly between wild-type and *Atcamta3* mutant plants (Figure 6), and thus further demonstrate that these genes may be the target genes of AtCAMTA3. Intriguingly, expression of these 10 genes altered differentially in *Atcamta3* mutant plants, eight of them (*SRS, CBP60G, CM2, ICE1, XLG2, RHL41/ZAT12, EDS1*, and *EDS16/ICS1*) were down-regulated while two *CBF* genes were up-regulated (Figure 6). The differential regulation of target gene expression by AtCAMTA3 has been reported previously. AtCAMTA3 directly binds to the *EDS1* and *NDR1* promoters and represses their expression during plant defense (Du et al., 2009; Nie et al., 2012), while it binds to the *CBF2* promoter and induces its expression during cold stress (Doherty et al., 2009). In this work, we also obtained similar results for *EDS1* and *CBF2* (Figure 6). In addition, our results indicate that similar to *CBF2, CBF1* could also be a target of *CAMTA3* which is positively regulated, while *SRS, CBP60G, CM2, ICE1, XLG2, RHL41/ZAT12*, and *EDS16/ICS1* are targets of *CAMTA3* which are negatively regulated.

Collectively, *AtCAMTA3* might target the predicted target genes to regulate biological processes such as biotic and abiotic stress responses. In addition, *AtCAMTA6* contained a CGCG element in its upstream sequence (Table S4). This indicates the direct interaction between *CAMTA* members. Confirmation of the interaction between AtCAMTAs and the predicted partners will provide insights into functional mechanisms of the CAMTA family in plants.

### Role and mechanism of *AtCAMTA3* in nonhost resistance

Plant disease resistance is classified into host resistance and nonhost resistance based on whether the plant is the host or nonhost of the inoculated pathogen. There have been several documents reporting the important role of CAMTA3 in host plant disease resistance against diverse pathogens including *Pst* DC3000, *B. cinerea, G. cichoracearum, M. grisea*, and *Xoo* (Galon et al., 2008; Du et al., 2009; Koo et al., 2009; Nie et al., 2012). However, as far as we know, no role of CAMTA3 in nonhost resistance has been reported so far. In the present study, we demonstrated that *Atcamta3* mutant plants exhibited enhanced HR and nonhost resistance to the bacterial pathogen *Xoo* (Figure 7), being the first report for the role of CAMTA3 in nonhost resistance.

The mechanism of CAMTA3 to regulate nonhost resistance remains unclear. Our recent study revealed that reactive oxygen species (ROS) such as H_2_O_2_ is indispensible for the *Xoo*-induced HR and nonhost resistance in *N. benthamiana* (Li et al., 2015). In this study, using the *Atcamta3* mutant, we demonstrated that *AtCAMTA3* negatively regulates the *Xoo*-induced and flg22-elicited H_2_O_2_ (Figure 8), revealing that *AtCAMTA3* negatively regulates nonhost resistance to *Xoo* via repressing ROS accumulation. This similar mechanism has been reported previously for *AtCAMTA3* to regulate host resistance to fungal and bacterial pathogens (Du et al., 2009; Nie et al., 2012). Moreover, we found significantly increased expression of the *EDS1, CBP60G*, and *NDR1* genes in both uninoculated and inoculated *Atcamta3* mutant plants compared with in wild-type plants (Figure 9). These genes might be involved in ROS accumulation. In addition, *CBP60G* were found to regulate salicylic acid (SA) biosynthesis triggered by microbe-associated molecular patterns (MAMPs) (Wang et al., 2011). Overexpression of *CBP60G* in Arabidopsis caused elevated SA accumulation, increased expression of the defense genes, and enhanced resistance to *Pseudomonas syringae* (Wan et al., 2012). Taken together, these results indicate that AtCAMTA3 targets and down-regulates expression of *EDS1, CBP60G*, and *NDR1* genes, which represses ROS accumulation, SA biosynthesis and signaling and expression of defense-related genes, and finally inhibits nonhost resistance to pathogens such as *Xoo*.

## Conclusions

The present study has identified and characterized 200 full-length *CAMTA* genes from 35 fully sequenced plant genomes. Approximately one fourth of the identified CAMTAs did not contain CDD database-recognizable TIG domain. This non-TIG class of CAMTAs is newly evolved from TIG class of CAMTAs through mutation of some key amino acids in the TIG domain in flowering land plants after divergence from non-flowering plants. The *CAMTA* genes are highly conserved in multicellular land plants but absent in unicellular eukaryotes, and are likely to have evolved from the fusion of two separate genes in the embryophyta ancestor lineage. Phylogenetic analysis classified CAMTA proteins into three major groups and nine distinct subgroups. All *CAMTA* genes from non-flowering plants were clustered into a single subgroup, while those from flowering plants fell into eight other subgroups, suggesting that the events leading to the expansion of the CAMTA family occurred in flowering plants. Generally, the gene structure is similar among the CAMTA orthologs in different species of flowering plants but dramatically different in paralogs of a given species. Gene duplication, intron invasion, enlargement, and turnover, and exon rearrangements and skipping clearly occurred during evolution of the CAMTA family. Thirty eight potential interactors were predicted for six Arabidopsis CAMTA proteins and about half of them contained at least one CGCG CAMTA-binding element in their promoter region. Ten predicted target genes of AtCAMTA3 exhibited differences in expression between *Atcamta3* mutants and wild-type plants, suggestting that these genes are likely to be the targets of AtCAMTA3. Functional analysis employing mutants revealed that Arabidopsis CAMTA3 negatively regulates nonhost resistance to bacterial pathogen *Xoo* probably via tuning *CBP60G, EDS1*, and *NDR1*-mediated ROS accumulation and SA-triggered immunity. Our results provide insights into the phylogeny of CAMTAs in plants and function of CAMTA3 in disease resistance. One of the next challenges will be elucidating the molecular regulatory mechanisms to activate and inactivate CAMTA proteins, such as revealing the possible coordinative roles of IQ and CaMB domains as well as CG-1 and TIG domains. Another challenge in the future is dissecting the molecular mechanisms of CAMTAs in regulating plant disease resistance, abiotic stress response and development, such as probing the possible specificity of association between CaM proteins and CAMTA proteins, and identifying other components to regulate CAMTAs and the downstream targets of CAMTAs.

## Author contributions

HR, Y-PX, and J-PM conducted the bioinformatics, phylogenetic, and evolutionary analyses. HR, JY, and J-PM carried out the gene expression and functional analysis. HR and Y-PX designed and analyzed all statistical data. X-ZC conceived of the study, and participated in its design and coordination. X-ZC and HR prepared the manuscript.

### Conflict of interest statement

The authors declare that the research was conducted in the absence of any commercial or financial relationships that could be construed as a potential conflict of interest.
