# Supplementary material for: Phylogeny of Plant CAMTAs and Role of AtCAMTAs in Nonhost Resistance to Xanthomonas oryzae pv. oryzae
Source: Front Plant Sci. 2016 Feb 29;7:177. doi: 10.3389/fpls.2016.00177 (PMC4770041; doi:10.3389/fpls.2016.00177)
Supplement: Supplementary file 1 [file Table1.PDF]

Supplementary Material

Phylogeny of plant CAMTAs and role of AtCAMTAs in nonhost resistance to *Xanthomonas oryzae* pv. *oryzae*

Hafizur Rahman[1], Juan Yang[1], You-Ping Xu[2], Jean-Pierre Munyampundu[1], Xin-Zhong Cai[1,3*]

[1] Institute of Biotechnology, College of Agriculture and Biotechnology, Zhejiang University, Hangzhou, China

[2] Center of Analysis and Measurement, Zhejiang University, Hangzhou, China

[3] State Key Laboratory of Rice Biology, Zhejiang University, Hangzhou, China

***Corresponding author:** Xin-Zhong Cai, Institute of Biotechnology, College of Agriculture and Biotechnology, Zhejiang University, 866 Yu Hang Tang Road, Hangzhou 310058, China.
E-mail: xzhcai@zju.edu.cn

Supplementary Tables

Table S1 | Summary of CAMTA proteins identified in this study.

Plant Family and Species	Gene Symbol	Protein ID No.	Gene Location	Protein Length (aa)	Protein Domains
**Funariaceae**					
Physcomitrella patens	PpCAMTA1	Phpat.003G141000.1.p	Chr03/ 23837629 - 23841096	1107	CG-1, TIG, Ank, IQ & CaMBD
	PpCAMTA2	Phpat.008G011300.1.p	Chr08/ 1359951 - 1364453	1129	CG-1, TIG, Ank, IQ & CaMBD
	PpCAMTA3	Phpat.004G019700.1.p	Chr04/ 3711302 - 3715938	1186	CG-1, TIG, Ank, IQ & CaMBD
**Selaginellaceae**					
Selaginella moellendorffii	SmCAMTA1	441667	Sfld 18: 708149-711976	982	CG-1, TIG, Ank, IQ & CaMBD
**Poaceae**					
Brachypodium distachyon	BdCAMTA1	Bradi1g21372.1	Bd1: 17182832 - 17189756	993	CG-1, TIG, Ank, IQ & CaMBD
	BdCAMTA2	Bradi1g60817.2	Bd1: 60186384 - 60194602	1020	CG-1, TIG, Ank, IQ & CaMBD
	BdCAMTA3	Bradi1g71810.2	Bd1: 69738566 - 69745888	1034	CG-1, TIG, Ank, IQ & CaMBD
	BdCAMTA4	Bradi2g59137.1	Bd2: 56912310 - 56917222	837	CG-1, TIG, Ank, IQ & CaMBD
	BdCAMTA5	Bradi1g27170.1	Bd1: 22256468 - 22265504	908	CG-1, Ank, IQ & CaMBD
	BdCAMTA6	Bradi5g08167.1	Bd5: 10739749 - 10747925	1022	CG-1, TIG, Ank, IQ & CaMBD
	BdCAMTA7	Bradi3g23800.1	Bd3: 23308658 - 23320967	1021	CG-1, TIG, Ank, IQ & CaMBD
Oryza sativa	OsCAMTA1	LOC_Os03g09100.1	Ch 03/ 4726139 - 4734011	1029	CG-1, TIG, Ank, IQ & CaMBD
	OsCAMTA2	LOC_Os10g22950.1	Ch10/ 11935626 - 11942908	1023	CG-1, TIG, Ank, IQ & CaMBD
	OsCAMTA3	LOC_Os07g43030.1	Ch 07/ 25776198 - 25783374	1026	CG-1, TIG, Ank, IQ & CaMBD
	OsCAMTA4	LOC_Os01g69910.1	Ch 01/ 40397315 - 40403249	878	CG-1, TIG, Ank, IQ & CaMBD
	OsCAMTA5	LOC_Os07g30774.1	Ch 07/ 18205276 - 18218190	927	CG-1, Ank, IQ & CaMBD
	OsCAMTA6	LOC_Os04g31900.1	Ch 04/ 19105902 - 19113570	1003	CG-1, TIG, Ank, IQ & CaMBD
Setaria italica	SiCAMTA1	Si028797m	Sfld 02: 46004982 - 46011911	1029	CG-1, TIG, Ank, IQ & CaMBD
	SiCAMTA2	Si034054m	Sfld 09: 43934615 - 43942014	1019	CG-1, TIG, Ank, IQ & CaMBD
	SiCAMTA3	Si034046m	Sfld 09: 22659263 - 22667628	1030	CG-1, TIG, Ank, IQ & CaMBD
	SiCAMTA4	Si009249m	Sfld 07: 17814717 - 17825117	1011	CG-1, TIG, Ank, IQ & CaMBD
	SiCAMTA5	Si028862m	Sfld 02: 40768574 - 40778086	913	CG-1, Ank, IQ & CaMBD
	SiCAMTA6	Si000282m	Sfld 05: 45113045 - 45119536	857	CG-1, TIG, Ank, IQ & CaMBD
	SiCAMTA7	Si034076m	Sfld 09: 54236883 - 54244124	994	CG-1, TIG, Ank, IQ & CaMBD
Zea mays	ZmCAMTA1	GRMZM2G341747_T01	5: 38258524 - 38268547	1020	CG-1, TIG, Ank, IQ & CaMBD
	ZmCAMTA2	GRMZM2G447551_T01	1: 19789491 - 19814550	1025	CG-1, TIG, Ank, IQ & CaMBD

	ZmCAMTA3	GRMZM2G431243_T01	9: 120356042 - 120364285	949	CG-1, TIG, Ank, IQ & CaMBD
	ZmCAMTA4	GRMZM2G171600_T01	3: 158895757 - 158901970	842	CG-1, TIG, Ank, IQ & CaMBD
	ZmCAMTA5	GRMZM2G032336_T01	7: 149602687 - 149621668	913	CG-1, Ank, IQ & CaMBD
	ZmCAMTA6	GRMZM2G152661_T01	10: 109572710 - 109580177	865	CG-1, TIG, Ank, IQ & CaMBD
	ZmCAMTA7	GRMZM2G143205_T01	2: 70334544 - 70342987	996	CG-1, TIG, Ank, IQ & CaMBD
Panicum virgatum	PvCAMTA1	Pavirv00064890m	sg0.contig12220: 2268 – 9130	1011	CG-1, TIG, Ank, IQ & CaMBD
	PvCAMTA2	Pavirv00018960m	sg0.contig49709: 193 – 5592	944	CG-1, TIG, Ank, IQ & CaMBD
	PvCAMTA3	Pavirv00029334m	sg0.contig07389: 1135 – 8207	1020	CG-1, TIG, Ank, IQ & CaMBD
	PvCAMTA4	Pavirv00022267m	sg0.contig03692: 4609 – 9400	851	CG-1, TIG, Ank, IQ & CaMBD
	PvCAMTA5	Pavirv00024367m	sg0.contig03408: 13315 – 19734	1016	CG-1, TIG, Ank, IQ & CaMBD
	PvCAMTA6	Pavirv00009054m	sg0.contig04988: 8707 – 16351	1021	CG-1, TIG, Ank, IQ & CaMBD
Sorghum bicolor	SbCAMTA1	Sobic.001G351700.1.p	Chr01/ 56917956 - 56927541	1024	CG-1, TIG, Ank, IQ & CaMBD
	SbCAMTA2	Sobic.002G380200.1.p	Chr02/ 73595589 - 73602889	1030	CG-1, TIG, Ank, IQ & CaMBD
	SbCAMTA3	Sobic.001G474600.1.p	Chr01/ 67483045 - 67490813	1034	CG-1, TIG, Ank, IQ & CaMBD
	SbCAMTA4	Sobic.003G407200.1.p	Chr03/ 71503317 - 71510562	845	CG-1, TIG, Ank, IQ & CaMBD
	SbCAMTA5	Sobic.002G306000.1.p	Chr02/ 68059313 - 68068718	914	CG-1, Ank, IQ & CaMBD
	SbCAMTA6	Sobic.006G051800.1.p	Chr06/ 38136976 - 38145091	988	CG-1, TIG, Ank, IQ & CaMBD
	SbCAMTA7	Sobic.K006800.1.p	super_12: 2468934 - 2477761	1021	CG-1, TIG, Ank, IQ & CaMBD
**Ranunculaceae**					
Aquilegia coerulea	AcCAMTA1	Aquca_003_00380.1	Sfld 03: 5404506 - 5418359	1037	CG-1, TIG, Ank, IQ & CaMBD
	AcCAMTA2	Aquca_010_00201.1	Sfld 10: 1207505 - 1215170	1083	CG-1, TIG, Ank, IQ & CaMBD
	AcCAMTA3	Aquca_008_00132.1	Sfld 08: 4061838 - 4073305	1092	CG-1, TIG, Ank, IQ & CaMBD
	AcCAMTA4	Aquca_040_00102.1	Sfld 40: 1680892 - 1696434	1037	CG-1, TIG, Ank, IQ & CaMBD
	AcCAMTA5	Aquca_003_00568.1	Sfld 03: 7508328 - 7520215	942	CG-1, TIG, Ank, IQ & CaMBD
**Scrophulariaceae**					
Mimulus guttatus v2.0	MgCAMTA1	Migut.B00609.1.p	scaffold_2: 2997766 - 3005404	994	CG-1, Ank, TIG, IQ & CaMBD
	MgCAMTA2	Migut.N00052.1.p	scaffold_14: 227208 - 235084	917	CG-1, Ank, IQ & CaMBD
	MgCAMTA3	Migut.G00093.1.p	scaffold_7: 649633 - 655986	931	CG-1, TIG, Ank, IQ & CaMBD
	MgCAMTA4	Migut.E00009.1.p	scaffold_5: 66596 - 73687	941	CG-1, TIG, Ank, IQ & CaMBD
**Solanaceae**					
Solanum lycopersicum	SlCAMTA1	JN558810	Ch 01/ 85225560-85236375	1037	CG-1, TIG, Ank, IQ & CaMBD
	SlCAMTA2	JN566050	Ch 01/ 53184734..53195240	939	CG-1, TIG, Ank & CaMBD
	SlCAMTA3	GU170838	Ch 04/ 53071230-53081628	1097	CG-1, Ank, IQ & CaMBD
	SlCAMTA4	JN566047	Ch 12/ 25868813-25886100	906	CG-1, TIG, Ank, IQ & CaMBD
	SlCAMTA5	JN566049	Ch 01/ 62496036-62529497	920	CG-1, TIG, Ank, IQ & CaMBD

	SlCAMTA6	JN566051	Ch 12/ 64863822-64873108	910	CG-1, TIG, Ank, IQ & CaMBD
	SlCAMTA7	JN566048	Ch 05/ 11602241-11617922	950	CG-1, TIG, Ank, IQ & CaMBD
Solanum tuberosum	StCAMTA1	XP_006351776.1	Unknown	1049	CG-1, TIG, Ank, IQ & CaMBD
	StCAMTA2	XP_006337966.1	Unknown	948	CG-1, TIG, Ank & CaMBD
	StCAMTA3	XP_006355338.1	Unknown	1101	CG-1, Ank, IQ & CaMBD
	StCAMTA4	XP_006341901.1	Unknown	973	CG-1, TIG, Ank, IQ & CaMBD
	StCAMTA5	XP_006355392.1	Unknown	923	CG-1, TIG, Ank, IQ & CaMBD
	StCAMTA6	XP_006349831.1	Unknown	915	CG-1, TIG, Ank, IQ & CaMBD
	StCAMTA7	XP_006352172.1	Unknown	962	CG-1, TIG, Ank, IQ & CaMBD
**Vitaceae**					
Vitis vinifera	VvCAMTA1	GSVIVG01017670001	Ch 05/ 2509331 - 2521854	1018	CG-1, TIG, Ank, IQ & CaMBD
	VvCAMTA2	GSVIVG01035027001	Ch 05/ 986562 - 999557	927	CG-1, Ank, IQ & CaMBD
	VvCAMTA3	GSVIVG01004860001	Ch 07 / 154711 - 169965	1243	CG-1, TIG, Ank, IQ & CaMBD
	VvCAMTA4	GSVIVG01010510001	Ch 01/ 21202170 - 21215480	968	CG-1, TIG, Ank, IQ & CaMBD
**Myrtaceae**					
Eucalyptus grandis	EgCAMTA1	Eucgr.I02632.1	Sfld 09: 37606276 - 37616247	993	CG-1, TIG, Ank, IQ & CaMBD
	EgCAMTA2	Eucgr.I02662.1	Sfld 09: 37828335 - 37838792	1056	CG-1, TIG, Ank, IQ & CaMBD
	EgCAMTA3	Eucgr.H04783.1	Sfld 08: 68188972 - 68205361	1047	CG-1, Ank, IQ & CaMBD
	EgCAMTA4	Eucgr.H04623.1	Sfld 08: 66330126 - 66342552	991	CG-1, TIG, Ank, IQ & CaMBD
**Rutaceae**					
Citrus clementina	CcCAMTA1	Ciclev10004234m	Sfld 09: 320448 - 327846	1017	CG-1, TIG, Ank, IQ & CaMBD
	CcCAMTA2	Ciclev10004273m	Sfld 09: 1000194 - 1008334	893	CG-1, TIG, Ank, IQ & CaMBD
	CcCAMTA3	Ciclev10024764m	Sfld 07: 20909144 - 20916408	1092	CG-1, Ank, IQ & CaMBD
	CcCAMTA4	Ciclev10030636m	Sfld 04: 21103928 - 21113129	973	CG-1, TIG, Ank, IQ & CaMBD
Citrus sinensis	CsCAMTA1	orange1.1g001759m	Sfld 00016: 1485810 - 1493604	1017	CG-1, TIG, Ank, IQ & CaMBD
	CsCAMTA2	orange1.1g001406m	Sfld 00336: 89141 - 102168	1083	CG-1, Ank, IQ & CaMBD
	CsCAMTA3	orange1.1g001365m	Sfld 00099: 53584 - 60886	1092	CG-1, Ank, IQ & CaMBD
	CsCAMTA4	orange1.1g044976m	Sfld 00003: 261176 - 269169	846	CG-1, TIG, Ank, IQ & CaMBD
	CsCAMTA5	orange1.1g002476m	Sfld 00016: 799153 - 807411	917	CG-1, TIG, Ank, IQ & CaMBD
**Malvaceae**					
Theobroma cacao	TcCAMTA1	Thecc1EG021468t1	Sfld 04: 33216390 - 33224051	966	CG-1, TIG, Ank, IQ & CaMBD
	TcCAMTA2	Thecc1EG007482t1	Sfld 02: 7230282 - 7240370	1064	CG-1, TIG, Ank, IQ & CaMBD
	TcCAMTA3	Thecc1EG001108t1	Sfld 01: 5267266 - 5277369	1085	CG-1, TIG, Ank, IQ & CaMBD
	TcCAMTA4	Thecc1EG008731t1	Sfld 02: 16450032 - 16457376	987	CG-1, TIG, Ank, IQ & CaMBD
	TcCAMTA5	Thecc1EG021327t2	Sfld 04: 32626014 - 32632466	907	CG-1, Ank, IQ & CaMBD

Gossypium raimondii	GrCAMTA1	Gorai.013G061100.1	Ch 13/ 6625467 - 6634301	1067	CG-1, TIG, Ank, IQ & CaMBD
	GrCAMTA2	Gorai.005G220600.1	Ch 05/ 60341564 - 60349789	1052	CG-1, TIG, Ank, IQ & CaMBD
	GrCAMTA3	Gorai.008G089900.1	Ch 08/ 21118330 - 21127475	1087	CG-1, TIG, Ank, IQ & CaMBD
	GrCAMTA4	Gorai.005G065700.1	Ch 05/ 7009778 - 7014890	910	CG-1, Ank, IQ & CaMBD
	GrCAMTA5	Gorai.011G198600.1	Ch 11/ 47994290 - 48001178	914	CG-1, Ank, IQ & CaMBD
	GrCAMTA6	Gorai.006G079000.1	Ch 06/ 30416003 - 30422953	907	CG-1, TIG, Ank, IQ & CaMBD
	GrCAMTA7	Gorai.011G204700.1	Ch 11/ 49581143 - 49586771	980	CG-1, TIG, Ank, IQ & CaMBD
**Caricaceae**					
Carica papaya	CpCAMTA1	evm.TU.supercontig_146.41	Super ctg 146: 197873 - 205506	729	CG-1, TIG, Ank, IQ & CaMBD
	CpCAMTA2	evm.TU.supercontig_929.1	Super ctg 929: 94 - 7974	855	CG-1, Ank, IQ & CaMBD
**Brassicaceae**					
Thellungiella halophila	ThCAMTA1	Thhalv10012552m	Sfld 02: 2965861 - 2971512	1020	CG-1, Ank, IQ & CaMBD
	ThCAMTA2	Thhalv10003564m	Sfld 06: 1357857 - 1364239	1063	CG-1, Ank, IQ & CaMBD
	ThCAMTA3	Thhalv10000026m	Sfld 15: 786488 - 792412	1041	CG-1, Ank, IQ & CaMBD
	ThCAMTA4	Thhalv10018053m	Sfld 09: 6910168 - 6917126	1051	CG-1, TIG, Ank, IQ & CaMBD
	ThCAMTA5	Thhalv10024342m	Sfld 01: 10880590 - 10885792	917	CG-1, TIG, Ank, IQ & CaMBD
	ThCAMTA6	Thhalv10020047m	Sfld 13: 3563891 - 3568471	861	CG-1, TIG, Ank, IQ & CaMBD
Brassica rapa	BrCAMTA1	Bra009382	A10: 16410948 - 16415731	1007	CG-1, Ank, IQ & CaMBD
	BrCAMTA2	Bra037769	A09: 3477081 - 3482359	1028	CG-1, Ank, IQ & CaMBD
	BrCAMTA3	Bra030248	A04: 9636736 - 9641698	1031	CG-1, Ank, IQ & CaMBD
	BrCAMTA4	Bra004096	A07: 16824946 - 16829722	973	CG-1, TIG, Ank, IQ & CaMBD
	BrCAMTA5	Bra038040	A08: 6984051 - 6988342	919	CG-1, TIG, Ank, IQ & CaMBD
	BrCAMTA6	Bra022188	A05: 19208838 - 19213220	850	CG-1, TIG, Ank, IQ & CaMBD
	BrCAMTA7	Bra038534	A09: 5149282 - 5153657	930	CG-1, Ank, IQ & CaMBD
	BrCAMTA8	Bra004217	A07: 17479235 - 17484080	997	CG-1, TIG, Ank, IQ & CaMBD
	BrCAMTA9	Bra034007	A02: 10255469 - 10259776	1012	CG-1, TIG, Ank, IQ & CaMBD
Capsella rubella	CrCAMTA1	Carubv10000120m	Sfld 06: 2913344 - 2919490	1028	CG-1, Ank, IQ & CaMBD
	CrCAMTA2	Carubv10025787m	Sfld 08: 12053631 - 12059994	1055	CG-1, Ank, IQ & CaMBD
	CrCAMTA3	Carubv10024738m	Sfld 04: 538133 - 543190	1032	CG-1, Ank, IQ & CaMBD
	CrCAMTA4	Carubv10022369m	Sfld 02: 8487158 - 8492469	1021	CG-1, TIG, Ank, IQ & CaMBD
	CrCAMTA5	Carubv10004090m	Sfld 07: 9653419 - 9659803	922	CG-1, TIG, Ank, IQ & CaMBD
	CrCAMTA6	Carubv10012963m	Sfld 03: 5911417 - 5917546	858	CG-1, TIG, Ank, IQ & CaMBD
Arabidopsis lyrata	AlCAMTA1	487754	Sfld 06: 3733150 - 3739335	997	CG-1, Ank, IQ & CaMBD
	AlCAMTA2	951243	Sfld 08: 21351394 - 21356987	1062	CG-1, Ank, IQ & CaMBD
	AlCAMTA3	481093	Sfld 04: 889534 - 894894	1031	CG-1, Ank, IQ & CaMBD

	AlCAMTA4	315745	Sfld 02: 12363954 - 12368866	1031	CG-1, TIG, Ank, IQ & CaMBD
	AlCAMTA5	355148	Sfld 07: 12351949 - 12356828	923	CG-1, TIG, Ank, IQ & CaMBD
	AlCAMTA6	318458	Sfld 03: 7236878 - 7241425	857	CG-1, TIG, Ank, IQ & CaMBD
Arabidopsis thaliana	AtCAMTA1	AT5G09410.3	Ch 05/ 2920827 - 2927420	1066	CG-1, Ank, IQ & CaMBD
	AtCAMTA2	AT5G64220.1	Ch 05/ 25686246 - 25692215	1050	CG-1, Ank, IQ & CaMBD
	AtCAMTA3	AT2G22300.1	Ch 02/ 9471388 - 9476646	1032	CG-1, Ank, IQ & CaMBD
	AtCAMTA4	AT1G67310.1	Ch 01/ 25198182 - 25203126	1016	CG-1, TIG, Ank, IQ & CaMBD
	AtCAMTA5	AT4G16150.1	Ch 04/ 9148059 - 9153292	923	CG-1, TIG, Ank, IQ & CaMBD
	AtCAMTA6	AT3G16940.1	Ch 03/ 5781775 - 5786280	845	CG-1, TIG, Ank, IQ & CaMBD
**Rosaceae**					
Fragaria vesca	FvCAMTA1	mrna29100.1-v1.0-hybrid	LG5: 18593938 - 18602314	1042	CG-1, TIG, Ank, IQ & CaMBD
	FvCAMTA2	mrna16294.1-v1.0-hybrid	LG1: 18483699 - 18490419	1086	CG-1, Ank, IQ & CaMBD
	FvCAMTA3	mrna16296.1-v1.0-hybrid	LG1: 18493288 - 18502816	1192	CG-1, TIG, Ank, IQ & CaMBD
	FvCAMTA4	mrna05159.1-v1.0-hybrid	LG4: 22655892 - 22662842	972	CG-1, TIG, Ank, IQ & CaMBD
Malus domestica	MdCAMTA1	MDP0000255517	MDC019345.551: 7362 - 15335	1122	CG-1, TIG, Ank, IQ & CaMBD
	MdCAMTA2	MDP0000320656	MDC015340.304: 22513 - 30165	952	CG-1, TIG, Ank, IQ & CaMBD
	MdCAMTA3	MDP0000290409	MDC011436.334: 48 - 11067	1153	CG-1, TIG, Ank, IQ & CaMBD
	MdCAMTA4	MDP0000219043	MDC022358.364: 4583 - 10254	1152	CG-1, TIG, Ank, IQ & CaMBD
	MdCAMTA5	MDP0000148340	MDC004313.539: 2580 - 8390	907	CG-1, Ank, IQ & CaMBD
	MdCAMTA6	MDP0000233860	MDC010246.376: 2071 - 8403	866	CG-1, Ank, IQ & CaMBD
	MdCAMTA7	MDP0000167705	MDC003086.270: 6137 - 12455	943	CG-1, Ank, IQ & CaMBD
	MdCAMTA8	MDP0000164873	MDC000464.247: 3873 - 9347	882	CG-1, Ank, IQ & CaMBD
Prunus persica	PpaCAMTA1	ppa000912m	Sfld 01: 8238644 - 8244925	964	CG-1, TIG, Ank, IQ & CaMBD
	PpaCAMTA2	ppa001493m	Sfld 01: 9174337 - 9182385	814	CG-1, Ank, IQ & CaMBD
	PpaCAMTA3	ppa000612m	Sfld 06: 17753322 - 17760978	1072	CG-1, TIG, Ank, IQ & CaMBD
	PpaCAMTA4	ppa000516m	Sfld 01: 22803671 - 22810283	1116	CG-1, TIG, Ank, IQ & CaMBD
**Cucurbitaceae**					
Cucumis sativus	CusCAMTA1	Cucsa.217940.1	Sfld 01658: 146208 - 159755	1092	CG-1, TIG, Ank, IQ & CaMBD
	CusCAMTA2	Cucsa.325250.1	Sfld 03127: 38216 - 50032	916	CG-1, TIG, Ank, IQ & CaMBD
	CusCAMTA3	Cucsa.349690.1	Sfld 03487: 911511 - 922170	899	CG-1, TIG, Ank, IQ & CaMBD
**Fabaceae**					
Glycine max	GmCAMTA1	Glyma05g24430.2	Gm05: 30613938 - 30624445	1088	CG-1, TIG, Ank, IQ & CaMBD
	GmCAMTA2	Glyma08g07680.2	Gm08: 5506034 - 5517351	1079	CG-1, TIG, Ank, IQ & CaMBD
	GmCAMTA3	Glyma05g31190.2	Gm05: 36357225 - 36365346	1122	CG-1, Ank, IQ & CaMBD

	GmCAMTA4	Glyma05g28090.2	Gm05: 33974971 - 33985589	983	CG-1, TIG, Ank, IQ & CaMBD
	GmCAMTA5	Glyma17g03510.1	Gm17: 2349225 - 2357540	922	CG-1, TIG, Ank, IQ & CaMBD
	GmCAMTA6	Glyma07g37090.2	Gm07: 42239449 - 42248514	921	CG-1, TIG, Ank, IQ & CaMBD
	GmCAMTA7	Glyma15g05900.2	Gm15: 4190636 - 4201836	1088	CG-1, TIG, Ank, IQ & CaMBD
	GmCAMTA8	Glyma08g14370.1	Gm08: 10445253 - 10453301	1102	CG-1, Ank, IQ & CaMBD
	GmCAMTA9	Glyma08g11080.1	Gm08: 8090234 - 8098571	966	CG-1, TIG, Ank, IQ & CaMBD
	GmCAMTA10	Glyma18g00840.2	Gm18: 394802 - 403759	962	CG-1, TIG, Ank, IQ & CaMBD
	GmCAMTA11	Glyma11g36930.2	Gm11: 38211262 - 38219718	910	CG-1, TIG, Ank, IQ & CaMBD
	GmCAMTA12	Glyma09g04310.2	Gm09: 3158659 - 3169052	911	CG-1, TIG, Ank, IQ & CaMBD
	GmCAMTA13	Glyma15g15350.1	Gm15: 11744864 - 11754888	911	CG-1, TIG, Ank, IQ & CaMBD
	GmCAMTA14	Glyma17g04310.2	Gm17: 2882121 - 2890411	999	CG-1, TIG, Ank, IQ & CaMBD
Phaseolus vulgaris	PhvCAMTA1	Phvul.006G206400.1	Ch 06/ 30900851 - 30911858	1076	CG-1, TIG, Ank, IQ & CaMBD
	PhvCAMTA2	Phvul.002G209300.1	Ch 02/ 36924678 - 36935363	1086	CG-1, TIG, Ank, IQ & CaMBD
	PhvCAMTA3	Phvul.002G256500.1	Ch 02/ 42310465 - 42317944	1105	CG-1, Ank, IQ & CaMBD
	PhvCAMTA4	Phvul.002G172800.1	Ch 02/ 32095502 - 32104715	987	CG-1, TIG, Ank, IQ & CaMBD
	PhvCAMTA5	Phvul.003G111900.1	Ch 03/ 28187173 - 28194709	922	CG-1, TIG, Ank, IQ & CaMBD
	PhvCAMTA6	Phvul.009G254500.1	Ch 05/ 36734077 - 36745918	911	CG-1, Ank, TIG, IQ & CaMBD
	PhvCAMTA7	Phvul.001G263000.1	Ch 01/ 51711829 - 51722211	966	CG-1, TIG, Ank, IQ & CaMBD
	PhvCAMTA8	Phvul.003G119800.1	Ch 03/ 29859348 - 29867209	997	CG-1, TIG, Ank, IQ & CaMBD
Medicago truncatula	MtCAMTA1	Medtr2g008840.1	Ch 02/ 1758073 - 1770948	1052	CG-1, TIG, Ank, IQ & CaMBD
	MtCAMTA2	Medtr4g121840.1	Ch 04/ 42045925 - 42052941	920	CG-1, TIG, Ank, IQ & CaMBD
	MtCAMTA3	Medtr2g034650.1	Ch 02/ 12077136 - 12083192	953	CG-1, TIG, Ank, IQ & CaMBD
	MtCAMTA4	Medtr3g085050.1	Ch 03/ 27701019 - 27710613	1081	CG-1, TIG, Ank, IQ & CaMBD
**Salicaceae**					
Populus trichocarpa	PtCAMTA1	Potri.010G153100.1	Ch 10/ 16079451 - 16086607	999	CG-1, TIG, Ank, IQ & CaMBD
	PtCAMTA2	Potri.007G093400.1	Ch 07/ 12024235 - 12037777	1091	CG-1, TIG, Ank, IQ & CaMBD
	PtCAMTA3	Potri.005G075100.1	Ch 05/ 5495668 - 5506028	1116	CG-1, TIG, Ank, IQ & CaMBD
	PtCAMTA4	Potri.001G057800.1	Ch 01/ 4383989 - 4392016	998	CG-1, TIG, Ank, IQ & CaMBD
	PtCAMTA5	Potri.010G141700.1	Ch 10/ 15323190 - 15330465	915	CG-1, Ank, IQ & CaMBD
	PtCAMTA6	Potri.008G107900.1	Ch 08/ 6848423 - 6856446	907	CG-1, TIG, Ank, IQ & CaMBD
	PtCAMTA7	Potri.003G170600.1	Ch 03/ 18050878 - 18059139	980	CG-1, TIG, Ank, IQ & CaMBD
**Linaceae**					
Linum usitatissimum	LuCAMTA1	Lus10003405	Sfld 644: 19853 - 26672	1103	CG-1, Ank, IQ & CaMBD
	LuCAMTA2	Lus10024044	Sfld 353: 205608 - 211044	959	CG-1, Ank, IQ & CaMBD
	LuCAMTA3	Lus10041704	Sfld 272: 1360328 - 1365986	973	CG-1, Ank, IQ & CaMBD

	LuCAMTA4	Lus10011352	Sfld 744: 129430 - 138462	1076	CG-1, TIG, Ank, IQ & CaMBD
	LuCAMTA5	Lus10036455	Sfld 57: 712304 - 719459	963	CG-1, TIG, Ank, IQ & CaMBD
	LuCAMTA6	Lus10037738	Sfld 196: 1328645 - 1333781	928	CG-1, TIG, Ank, IQ & CaMBD
	LuCAMTA7	Lus10003119	Sfld 1847: 2666 - 6412	900	CG-1, TIG, Ank, IQ & CaMBD
	LuCAMTA8	Lus10016873	Sfld 153: 370083 - 375177	901	CG-1, TIG, Ank, IQ & CaMBD
**Euphorbiaceae**					
Ricinus communis	RcCAMTA1	30063.m001397	30063: 4574 - 14129	1019	CG-1, Ank, IQ & CaMBD
	RcCAMTA2	29889.m003269	29889: 139260 - 146822	918	CG-1, Ank, IQ & CaMBD
	RcCAMTA3	29889.m003372	29889: 732936 - 740559	999	CG-1, TIG, Ank, IQ & CaMBD
	RcCAMTA4	29929.m004634	29929: 796679 - 804176	924	CG-1, TIG, Ank, IQ & CaMBD
Manihot esculenta	MeCAMTA1	cassava4.1_023110m	Sfld 08828: 16407 - 23168	977	CG-1, TIG, Ank, IQ & CaMBD
	MeCAMTA2	cassava4.1_000687m	Sfld 10292: 72404 - 87710	1073	CG-1, Ank, IQ & CaMBD
	MeCAMTA3	cassava4.1_000675m	Sfld 08265: 767198 - 775785	1079	CG-1, TIG, Ank, IQ & CaMBD
	MeCAMTA4	cassava4.1_000943m	Sfld 03219: 492033 - 499806	991	CG-1, TIG, Ank, IQ & CaMBD
	MeCAMTA5	cassava4.1_030577m	Sfld 01945: 191543 - 199092	861	CG-1, Ank, IQ & CaMBD

Abbreviations used in table: CG-1 (CG-1 domain), Ank (Ankyrin repeat domain), IQ (IQ motif) and CaMBD (Calmodulin binding domain).

Table S2 | Distribution of CAMTA genes with various introns.

CAMTA gene types based on introns	Distribution of different types of CAMTA genes									Total	%
	Ia	Ib	IIa	IIb	IIIa	IIIb	IIIc	IIId	IIIe		
12	32	5	20	1	1	1	24	20	14	118	59.0
11	8	0	10	6	0	10	2	3	4	43	21.5
>12	2	0	1	0	0	2	3	0	6	14	7.0
<11	5	0	3	6	3	5	0	1	2	25	12.5
Total	47	5	34	13	4	18	29	24	26	200	100

Table S3 | Introns of all CAMTA genes identified in 35 plant species.

Plant Species	Total No. of Genes	Gene Symbol	No. of Introns
Physcomitrella patens	3	PpCAMTA1	1
		PpCAMTA2	1
		PpCAMTA3	0
Selaginella moellendorffii	1	SmCAMTA1	12
Brachypodium distachyon	7	BdCAMTA1	11
		BdCAMTA2	12
		BdCAMTA3	12
		BdCAMTA4	10
		BdCAMTA5	12
		BdCAMTA6	11
		BdCAMTA7	12
Oryza sativa	6	OsCAMTA1	12
		OsCAMTA2	12
		OsCAMTA3	12
		OsCAMTA4	10
		OsCAMTA5	12
		OsCAMTA6	11
Setaria italica	7	SiCAMTA1	12
		SiCAMTA2	12
		SiCAMTA3	12
		SiCAMTA4	11
		SiCAMTA5	12
		SiCAMTA6	10
		SiCAMTA7	11
Zea mays	7	ZmCAMTA1	12
		ZmCAMTA2	12
		ZmCAMTA3	12
		ZmCAMTA4	10
		ZmCAMTA5	12
		ZmCAMTA6	12
		ZmCAMTA7	11
Panicum virgatum	6	PvCAMTA1	11
		PvCAMTA2	9
		PvCAMTA3	12
		PvCAMTA4	8
		PvCAMTA5	11
		PvCAMTA6	12
Sorghum bicolor	7	SbCAMTA1	12
		SbCAMTA2	12

		SbCAMTA3	12
		SbCAMTA4	10
		SbCAMTA5	12
		SbCAMTA6	11
		SbCAMTA7	12
Aquilegia coerulea	5	AcCAMTA1	11
		AcCAMTA2	12
		AcCAMTA3	12
		AcCAMTA4	11
		AcCAMTA5	11
Mimulus guttatus v2.0	4	MgCAMTA1	12
		MgCAMTA2	12
		MgCAMTA3	12
		MgCAMTA4	11
Solanum lycopersicum	7	SlCAMTA1	13
		SlCAMTA2	10
		SlCAMTA3	12
		SlCAMTA4	11
		SlCAMTA5	12
		SlCAMTA6	12
		SlCAMTA7	12
Solanum tuberosum	7	StCAMTA1	13
		StCAMTA2	11
		StCAMTA3	12
		StCAMTA4	11
		StCAMTA5	12
		StCAMTA6	12
		StCAMTA7	11
Vitis vinifera	4	VvCAMTA1	11
		VvCAMTA2	12
		VvCAMTA3	12
		VvCAMTA4	12
Eucalyptus grandis	4	EgCAMTA1	11
		EgCAMTA2	12
		EgCAMTA3	12
		EgCAMTA4	12
Citrus clementina	4	CcCAMTA1	11
		CcCAMTA2	12
		CcCAMTA3	12
		CcCAMTA4	12
Citrus sinensis	5	CsCAMTA1	11

		CsCAMTA2	12
		CsCAMTA3	12
		CsCAMTA4	9
		CsCAMTA5	12
Theobroma cacao	5	TcCAMTA1	11
		TcCAMTA2	12
		TcCAMTA3	12
		TcCAMTA4	11
		TcCAMTA5	12
Gossypium raimondii	7	GrCAMTA1	12
		GrCAMTA2	12
		GrCAMTA3	12
		GrCAMTA4	12
		GrCAMTA5	12
		GrCAMTA6	12
		GrCAMTA7	10
Carica papaya	2	CpCAMTA1	6
		CpCAMTA2	13
Thellungiella halophila	6	ThCAMTA1	12
		ThCAMTA2	12
		ThCAMTA3	13
		ThCAMTA4	12
		ThCAMTA5	12
		ThCAMTA6	11
Brassica rapa	9	BrCAMTA1	12
		BrCAMTA2	12
		BrCAMTA3	11
		BrCAMTA4	12
		BrCAMTA5	12
		BrCAMTA6	11
		BrCAMTA7	9
		BrCAMTA8	12
		BrCAMTA9	12
Capsella rubella	6	CrCAMTA1	12
		CrCAMTA2	12
		CrCAMTA3	13
		CrCAMTA4	12
		CrCAMTA5	12
		CrCAMTA6	11
Arabidopsis lyrata	6	AlCAMTA1	12
		AlCAMTA2	12

		AlCAMTA3	13
		AlCAMTA4	12
		AlCAMTA5	12
		AlCAMTA6	11
Arabidopsis thaliana	6	AtCAMTA1	15
		AtCAMTA2	12
		AtCAMTA3	13
		AtCAMTA4	12
		AtCAMTA5	12
		AtCAMTA6	9
Fragaria vesca	4	FvCAMTA1	13
		FvCAMTA2	11
		FvCAMTA3	15
		FvCAMTA4	12
Malus domestica	8	MdCAMTA1	12
		MdCAMTA2	13
		MdCAMTA3	13
		MdCAMTA4	11
		MdCAMTA5	11
		MdCAMTA6	11
		MdCAMTA7	13
		MdCAMTA8	12
Prunus persica	4	PpaCAMTA1	9
		PpaCAMTA2	9
		PpaCAMTA3	11
		PpaCAMTA4	11
Cucumis sativus	3	CusCAMTA1	12
		CusCAMTA2	12
		CusCAMTA3	10
Glycine max	14	GmCAMTA1	12
		GmCAMTA2	12
		GmCAMTA3	12
		GmCAMTA4	12
		GmCAMTA5	12
		GmCAMTA6	12
		GmCAMTA7	12
		GmCAMTA8	12
		GmCAMTA9	12
		GmCAMTA10	12
		GmCAMTA11	12
		GmCAMTA12	12
		GmCAMTA13	12

		GmCAMTA14	11
Phaseolus vulgaris	8	PhvCAMTA1	12
		PhvCAMTA2	12
		PhvCAMTA3	12
		PhvCAMTA4	12
		PhvCAMTA5	12
		PhvCAMTA6	12
		PhvCAMTA7	12
		PhvCAMTA8	11
Medicago truncatula	4	MtCAMTA1	11
		MtCAMTA2	12
		MtCAMTA3	12
		MtCAMTA4	15
Populus trichocarpa	7	PtCAMTA1	11
		PtCAMTA2	12
		PtCAMTA3	12
		PtCAMTA4	12
		PtCAMTA5	11
		PtCAMTA6	12
		PtCAMTA7	12
Linum usitatissimum	8	LuCAMTA1	12
		LuCAMTA2	9
		LuCAMTA3	11
		LuCAMTA4	11
		LuCAMTA5	10
		LuCAMTA6	12
		LuCAMTA7	9
		LuCAMTA8	10
Ricinus communis	4	RcCAMTA1	11
		RcCAMTA2	12
		RcCAMTA3	12
		RcCAMTA4	11
Manihot esculenta	5	MeCAMTA1	10
		MeCAMTA2	12
		MeCAMTA3	12
		MeCAMTA4	12
		MeCAMTA5	10

Table S4 | 38 predicted functional partner proteins mapped with STRING database.

Protein ID	Description	Function	Interaction with specific AtCAMTA and confidence score						CGCG *cis*-element and position
			CAMTA1	CAMTA2	CAMTA3	CAMTA4	CAMTA5	CAMTA6	
AT1G11870.2	SRS (Seryl-tRNA Synthetase)	Unknown	√ (0.722)		√ (0.721)	√ (0.548)	√ (0.820)	√ (0.548)	GCGCGG (-951 ~ -946)
AT1G67530.1	Armadillo/beta-catenin repeat family protein	Ubiquitin-protein ligase activity	√ (0.887)						
AT5G01820.1	CIPK14 (CBL-Interacting Protein Kinase 14)	ATP binding, Kinase activity, protein binding, protein serine/threonine kinase activity			√ (0.829)	√ (0.638)	√ (0.769)	√ (0.638)	
AT1G73805.1	SARD1 (SAR Deficient 1)	Calmodulin binding, sequence-specific DNA binding transcription factor activity			√ (0.828)				
AT4G11640.1	SR (Serine Racemase)	ATP binding, D-serine ammonia-lyase activity, L-serine ammonia-lyase activity,metal ion binding, serine racemase activity					√ (0.820)		
AT5G26920.1	CBP60G (Calmodulin-binding protein 60-Like.G)	Calmodulin binding, sequence-specific DNA binding activity			√ (0.820)				ACGCGT (-1157 ~ -1152)
AT1G75080.1	BZR1 (Brassinazole-Resistant 1)	Protein binding, sequence-specific DNA binding transcription factor activity					√ (0.790)		ACGCGG (-1141 ~ -1136)
AT5G46830.1	NIG1 (NACL-inducible gene 1)	DNA binding, calcium ion binding	√ (0.727)						
AT2G37025.1	TRFL8 (TRF-like 8)	DNA binding, chromatin binding, regulation of transcription	√ (0.726)						
AT5G61900.1	BON1	Calcium-dependent phospholipid binding			√ (0.710)				
AT1G76510.3	ARID/BRIGHT DNA-binding domain-containing protein	DNA binding, sequence-specific DNA binding transcription factor activity		√ (0.648)					

AT5G10870.1	CM2 (Chorismate mutase 2)	Chorismate mutase activity			√ (0.647)		ACGCGG (-927 ~ -922) ACGCGT (-1220 ~ -1215)
AT3G23000.1	CIPK7 (CBL-Interacting Protein Kinase 7)	Kinase activity, protein binding, protein serine/threonine kinase activity				√ (0.643)	
AT5G54590.2	CRLK1 (Calcium/calmodulin-regulated receptor-like kinase 1)	ATP binding, calmodulin binding	√ (0.624)				GCGCGT (-341 ~ -336)
AT4G25480.1	DREB1A/CBF3 (Dehydration Response Element B1A)	DNA binding, sequence-specific DNA binding transcription factor activity			√ (0.595)		
AT4G35580.2	NTL9 (NAC transcription factor-like 9)	DNA binding, calmodulin binding, sequence-specific DNA binding transcription factor activity			√ (0.589)		
AT3G26744.4	ICE1 (Inducer of CBF Expression 1)	Protein binding, sequence-specific DNA binding transcription factor activity			√ (0.587)		ACGCGT (-561 ~ -556)
AT4G34390.1	XLG2 (Extra-large GTP-binding protein 2)	G-protein beta/gamma-subunit complex binding, G-protein coupled receptor binding, GTP binding, protein binding, signal transducer activity			√ (0.584)		CCGCGT (-493 ~ -488)
AT5G52830.1	WRKY27	Sequence-specific DNA binding transcription factor activity	√ (0.580)				
AT4G33280.1	AP2/B3-like transcriptional factor family protein	DNA binding, sequence-specific DNA binding transcription factor activity		√ (0.580)			
AT4G13640.2	UNE16 (Unfertilized embryo sac 16)	Chromatin binding, sequence-specific DNA binding transcription factor activity		√ (0.578)			
AT1G43860.1	Unknown protein	RNA binding, sequence-specific DNA binding transcription factor activity		√ (0.578)			

AT1G74660.1	MIF1 (Mini Zinc Finger 1)	Metal ion binding, protein homodimerization activity, sequence-specific DNA binding transcription factor activity		√ (0.576)					
AT2G41010.1	CAMBP25 (Calmodulin Binding Protein 25)	Calmodulin Binding	√ (0.573)						ACGCGG (-137 ~ -132) CCGCGT (-454 ~ -449) ACGCGT (-1106 ~ -1101)
AT4G14770.1	TCX2 (TESMIN/TSO1-like CXC 2)	Metal ion binding, sequence-specific DNA binding transcription factor activity		√ (0.572)					
AT5G59820.1	RHL41/ZAT12 (Responsive to High Light 41)	Nucleic acid binding, zinc ion binding			√ (0.569)				CCGCGC (-513 ~ -508) ACGCGT (-1056 ~ -1051)
AT5G63840.1	RSW3 (Radial Swelling 3)	Carbohydrate binding, glucan 1,3-alpha-glucosidase activity, glucosidase activity, hydrolase activity		√ (0.554)		√ (0.554)	√ (0.554)	√ (0.554)	CCGCGT (-322 ~ -317)
AT3G23640.1	HGL1 (Heteroglycan Glucosidase 1)	Carbohydrate binding, hydrolase activity		√ (0.554)		√ (0.554)	√ (0.554)	√ (0.554)	
AT4G25490.1	CBF1 (C-Repeat/DRE Binding Factor 1)	DNA binding, sequence-specific DNA binding transcription factor activity			√ (0.550)				CCGCGG (-1118 ~ -1113)
AT1G22930.1	T-complex protein 11	Unknown						√ (0.547)	
AT1G05690.1	BT3 (BTB and TAZ domain protein 3)	Calmodulin binding, histone acetyltransferase activity, transcription cofactor activity, zinc ion binding	√ (0.547)						CCGCGG (-1398 ~ -1393)
AT3G50410.1	OBP1 (OBF binding protein 1)	DNA binding, protein binding, transcription regulatory region DNA binding		√ (0.544)					

AT4G25470.1	CBF2 (C-Repeat/DRE Binding Factor2)	DNA binding, sequence-specific DNA binding transcription factor activity		√ (0.543)			CCGCGT (224 ~ -219)
AT3G48090.1	EDS1 (Enhanced Disease Susceptibility 1)	Lipase activity, protein binding, protein homodimerization activity, signal transducer activity, triglyceride lipase activity		√ (0.543)			ACGCGT (-746 ~ -741)
AT1G74710.2	EDS16/ ICS1 (Enhanced disease susceptibility to *Erysiphe orontii* 16)/Isochorismate synthase 1	Isochorismate synthase activity		√ (0.542)			CCGCGT (-1577 ~ -1572)
AT2G42380.2	BZIP34 (Basic helix-loop-helix domain-containing protein 34)	Protein binding, sequence-specific DNA binding transcription factor activity	√ (0.515)				
AT3G16940.1	AtCAMTA6 (Calmodulin-binding transcription activator 6)	Calmodulin binding			√ (0.507)		CCGCGG (-748 ~ -743)
AT1G67310.1	AtCAMTA4 (Calmodulin-binding transcription activator 4)	Calmodulin binding				√ (0.507)	

Table S5 | Primers used for RT-PCR analysis in this study.

Target Gene	Primer Name	Sequence (5’-3’)
EDS1	EDS1-F	GTCTACGCTCAATGACCTTGGAGTG
	EDS1-R	CATTTTTATGGGCTTGACACTTTGG
CBP60g	CBP60g-F	TCGTGGACGCCACCACAAACA
	CBP60g-R	TCAGCGTTCAGCGGCACGAG
EDS16	ICS1-F	TATCTCCGGCAGCCGCCACT
	ICS1-R	ACGCCGGAGGAAAACGACGG
CBF1	CBF1-F	TCCAAAGCGACACGTCACCATCTC
	CBF1-R	CCGCCGTCTGTTCAATGGAATCAT
CBF2	CBF2-F	GACGTGTCCTTATGGAGCTATTAAAA
	CBF2-R	TTACCATTTACATTCGTTTCTCACAAC
RLH41	RLH41-F	CCTTAGGAGGTCACCGTGC
	RLH41-R	CAAGCCACTCTCTTCCCACT
SRS	SRS-F	CCATCTGAACCGCCTCAGA
	SRS-R	AGCAAACACACCATCATCCTT
CM2	CM2-F	TCGCGCTCAGGATAGAGAGG
	CM2-R	TCATCGCCATAGCCAGAGTTG
XLG2	XLG2-F	AGGTCCTCTTATTGCTAATGTG
	XLG2-R	GTCTCTTGGTATCCTTAGTCA
ICE1	ICE1-F	CTCAATGTTCCTTCTTCTGCA
	ICE1-R	CTCAAATCCCTGTTCCCCAA
NDR1	NDR1-F	CTTTTCTTATGGCTTAGTCTCCGTG
	NDR1-R	ATCTTGGTCGTGTTGATGGTGG
PR1	PR1-F	AAAGCTCAAGATAGCCCACA
	PR1-R	AGCCTTCTCGCTAACCCACA
CAMTA3	CAMTA3-F	GACGCGGCTCGGTCTCCTCAAG
	CAMTA3-R	AGCTGTGGAACTTCCATGCTG
Actin8	Actin8_F	CGAGGCTCCTCTTAACCCAAA
	Actin8_R	GGCACAGTGTGAGACACACCA
