# Supplementary material for: Phylogeny of Plant CAMTAs and Role of AtCAMTAs in Nonhost Resistance to Xanthomonas oryzae pv. oryzae
Source: Front Plant Sci. 2016 Feb 29;7:177. doi: 10.3389/fpls.2016.00177 (PMC4770041; doi:10.3389/fpls.2016.00177)
Supplement: Supplementary file 2 [file Image1.PDF]

Supplementary Material

Phylogeny of plant CAMTAs and role of AtCAMTAs in nonhost resistance to *Xanthomonas oryzae* pv. *oryzae*

Hafizur Rahman[1], Juan Yang[1], You-Ping Xu[2], Jean-Pierre Munyampundu[1], Xin-Zhong Cai[1,3*]

[1] Institute of Biotechnology, College of Agriculture and Biotechnology, Zhejiang University, Hangzhou, China

[2] Center of Analysis and Measurement, Zhejiang University, Hangzhou, China

[3] State Key Laboratory of Rice Biology, Zhejiang University, Hangzhou, China

***Corresponding author:** Xin-Zhong Cai, Institute of Biotechnology, College of Agriculture and Biotechnology, Zhejiang University, 866 Yu Hang Tang Road, Hangzhou 310058, China.
E-mail: xzhcai@zju.edu.cn

Supplementary Figures

A

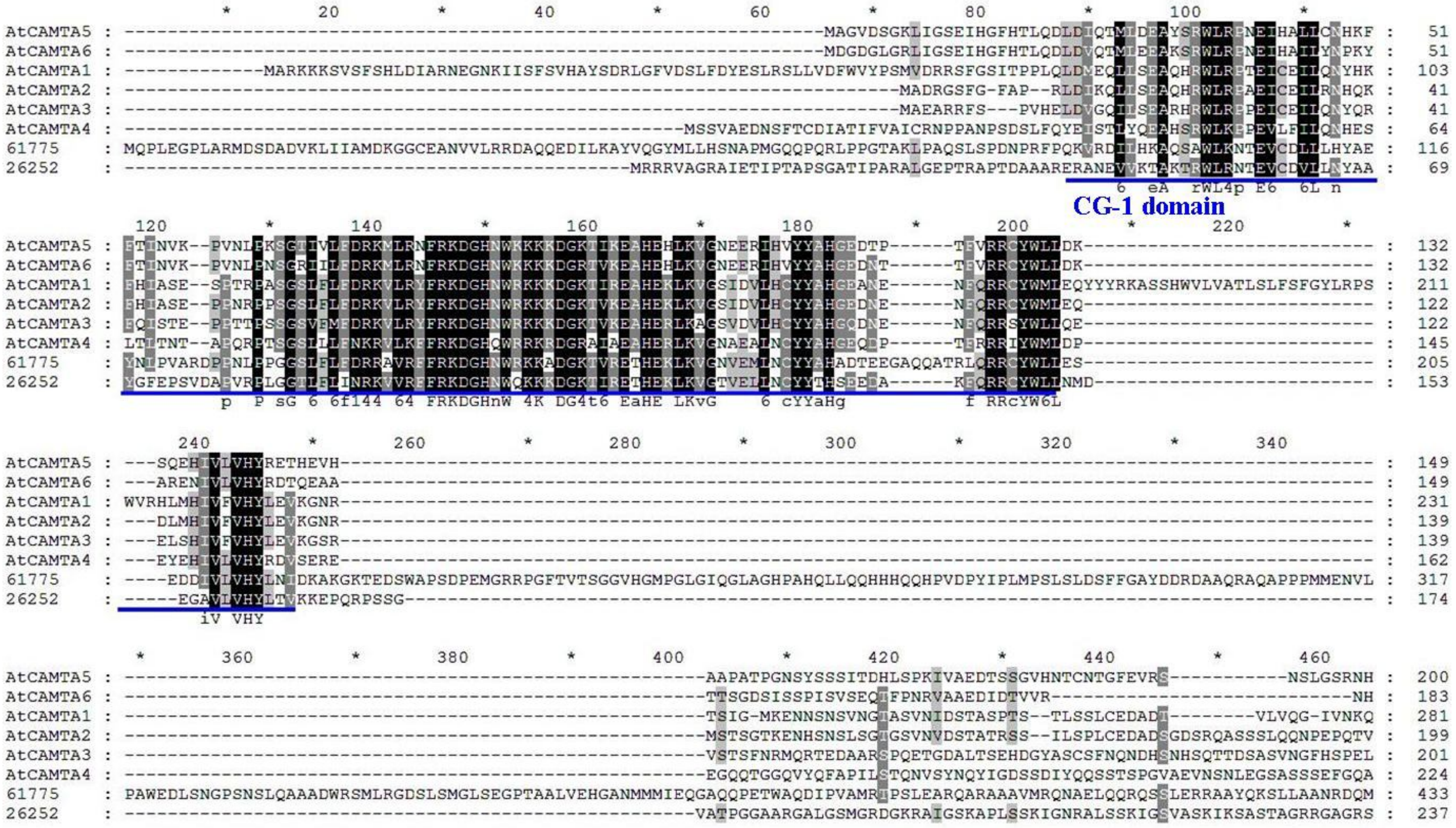

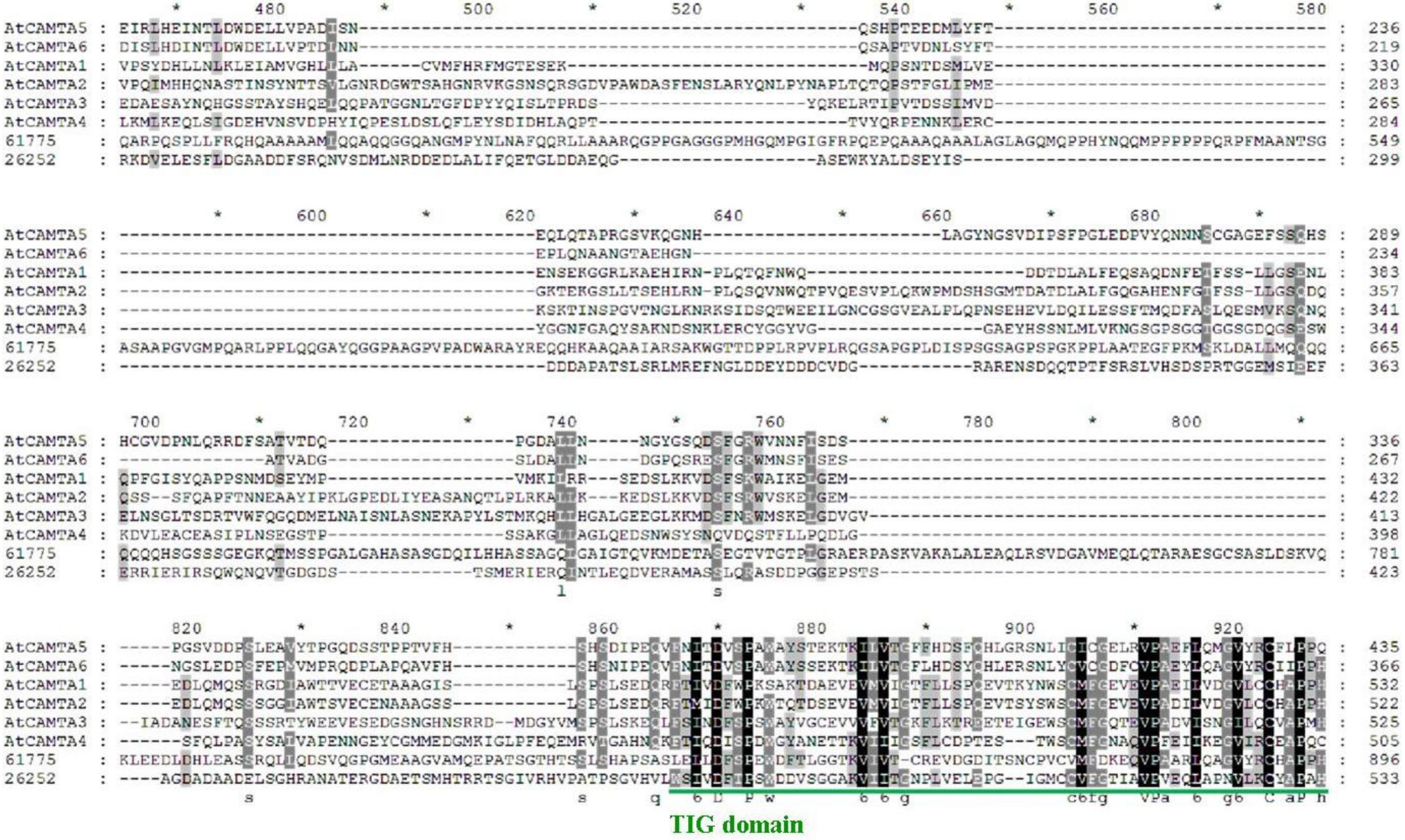
AtCAMTA5
AtCAMTA6
AtCAMTA1
AtCAMTA2
AtCAMTA3
AtCAMTA4
61775
26252
TIG domain

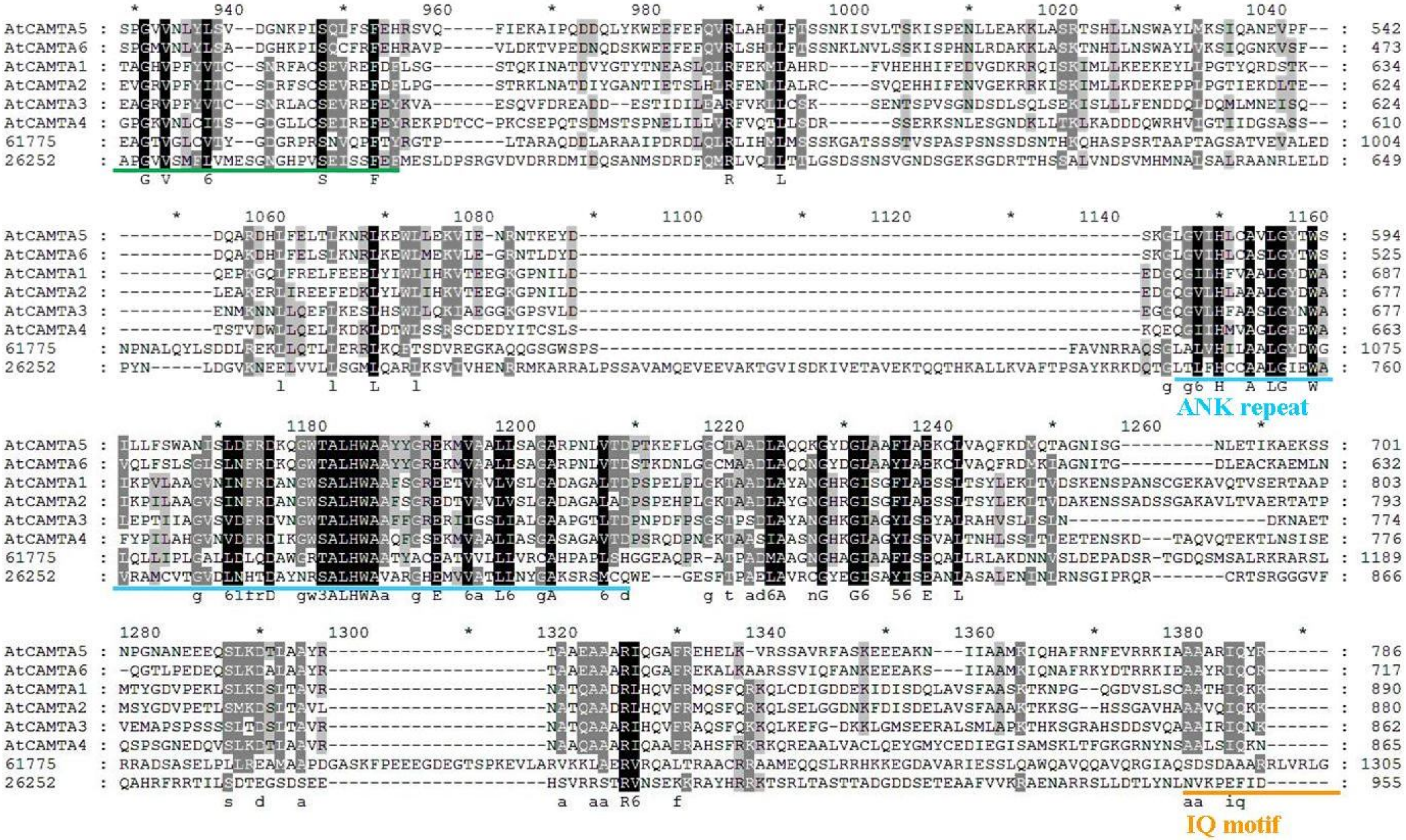
ANK repeat
IQ motif

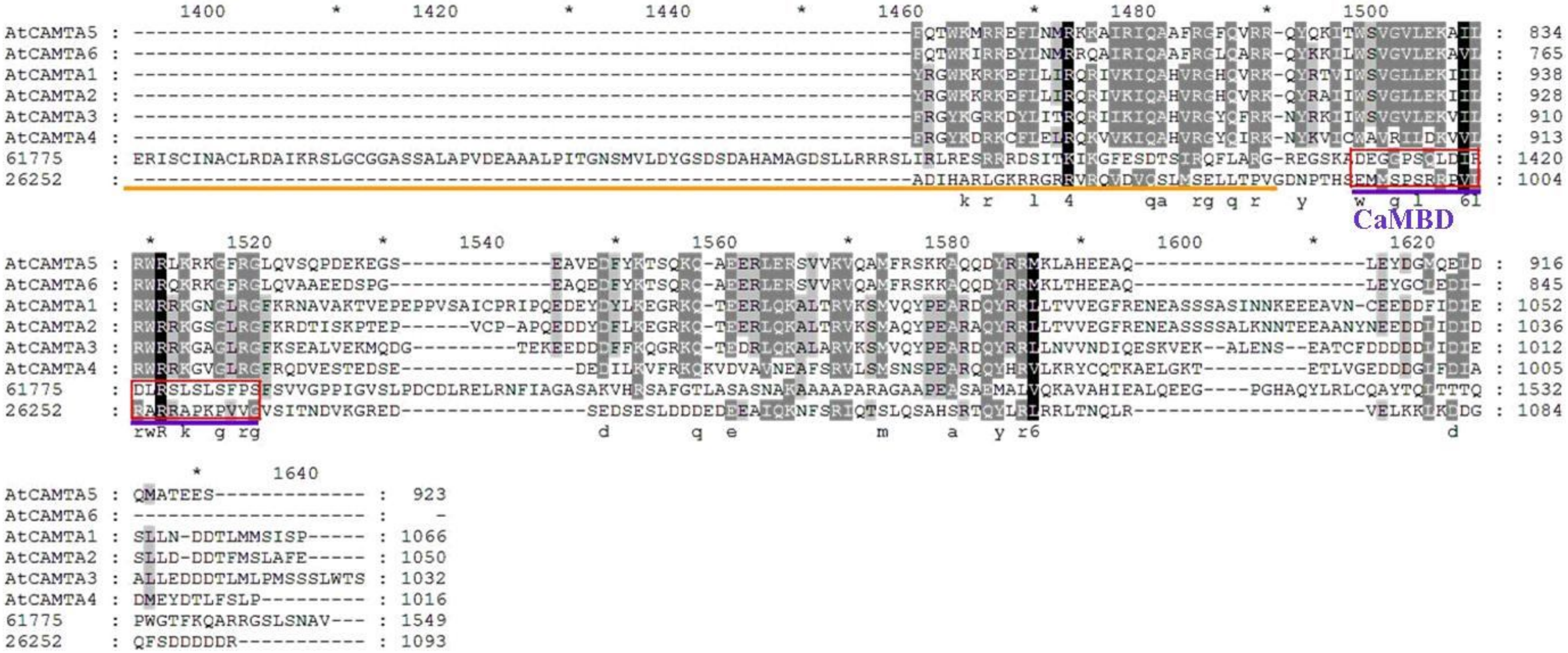
CaMBD
AtCAMTA5
AtCAMTA6
AtCAMTA1
AtCAMTA2
AtCAMTA3
AtCAMTA4
61775
26252

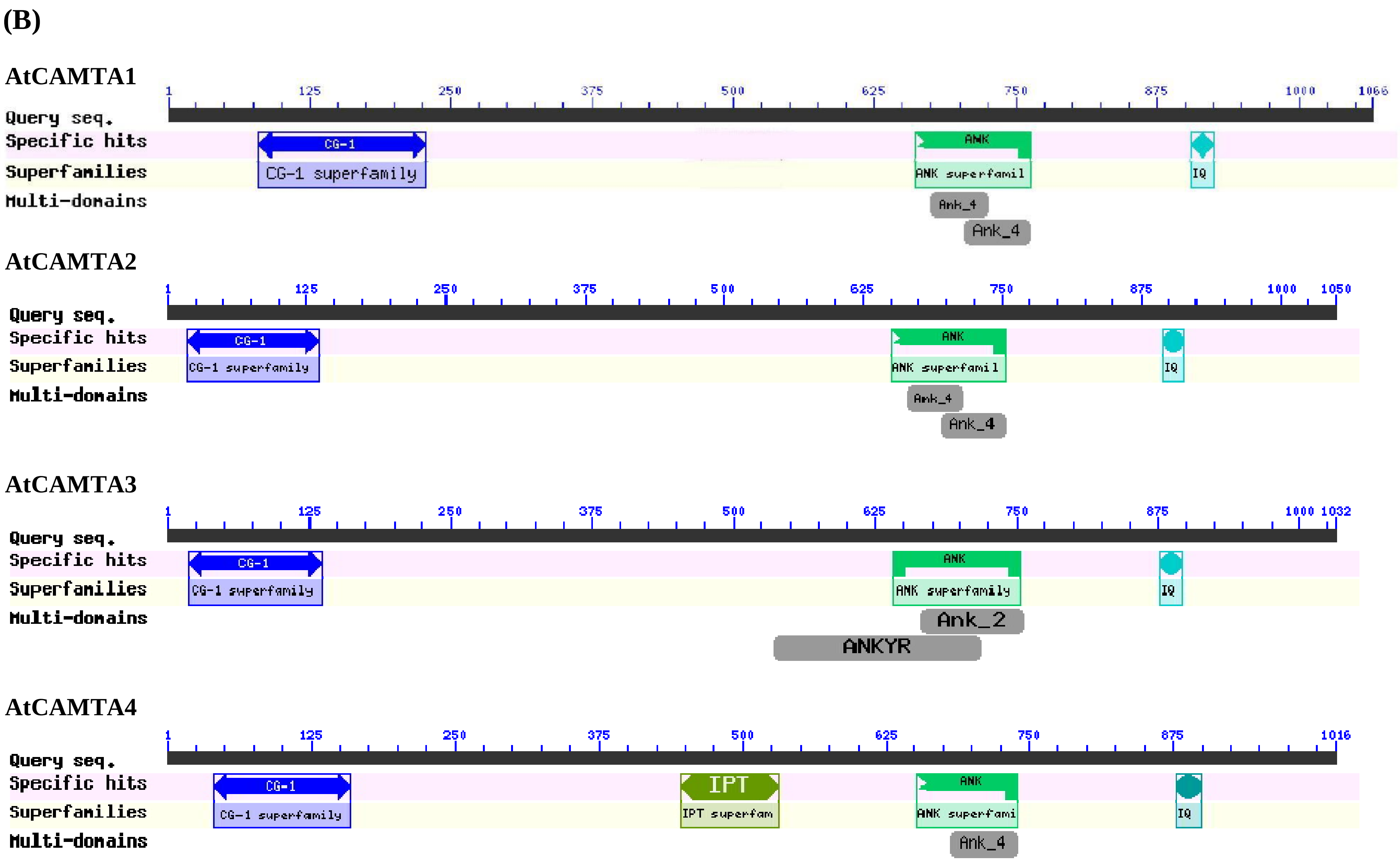
(B)
AtCAMTA1
Query seq.
Specific hits
Superfamilies
Multi-domains
CG-1
CG-1 superfamily
ANK
ANK superfamil
IQ
Ank_4
Ank_4
AtCAMTA2
CG-1
CG-1 superfamily
ANK
ANK superfamil
IQ
Ank_4
Ank_4
AtCAMTA3
CG-1
CG-1 superfamily
ANK
ANK superfamily
IQ
Ank_2
ANKYR
AtCAMTA4
CG-1
CG-1 superfamily
IPT
IPT superfam
ANK
ANK superfami
IQ
Ank_4

AtCAMTA5

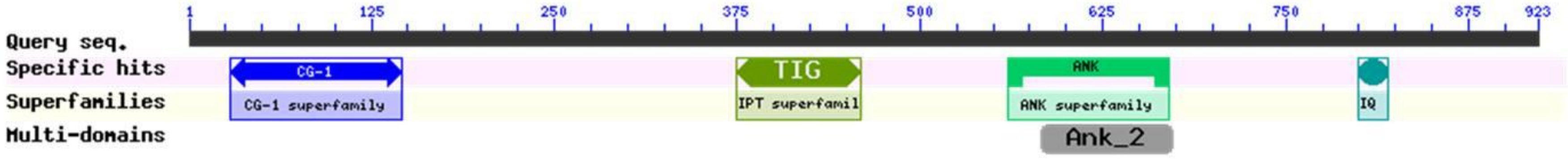

AtCAMTA6

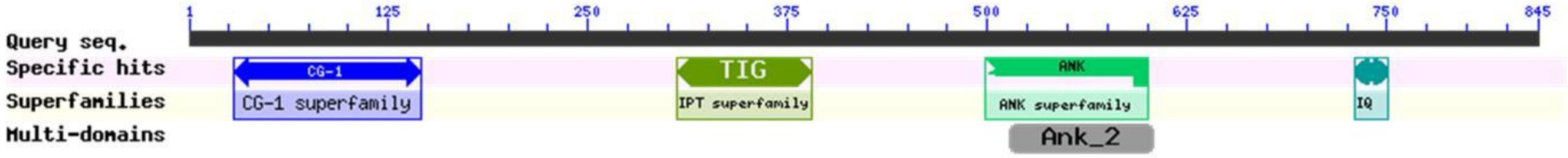

Ostreococcus lucimarinus **(Protein ID 26252)**

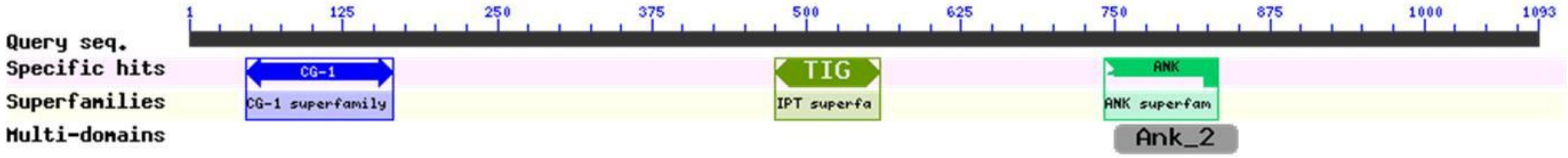

Coccomyxa subellipsoidea **(Protein ID 61775)**

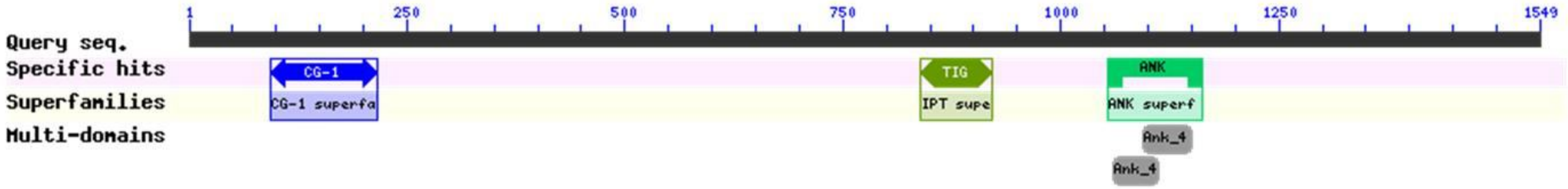

Figure S1 | Domain organization of two CAMTA like proteins in algae and six Arabidopsis CAMTAs. Multiple sequence alignment **(A)** and domain composition **(B)** of CAMTA and CAMTA like proteins. The domain composition analysis was performed using NCBI-CDD program.

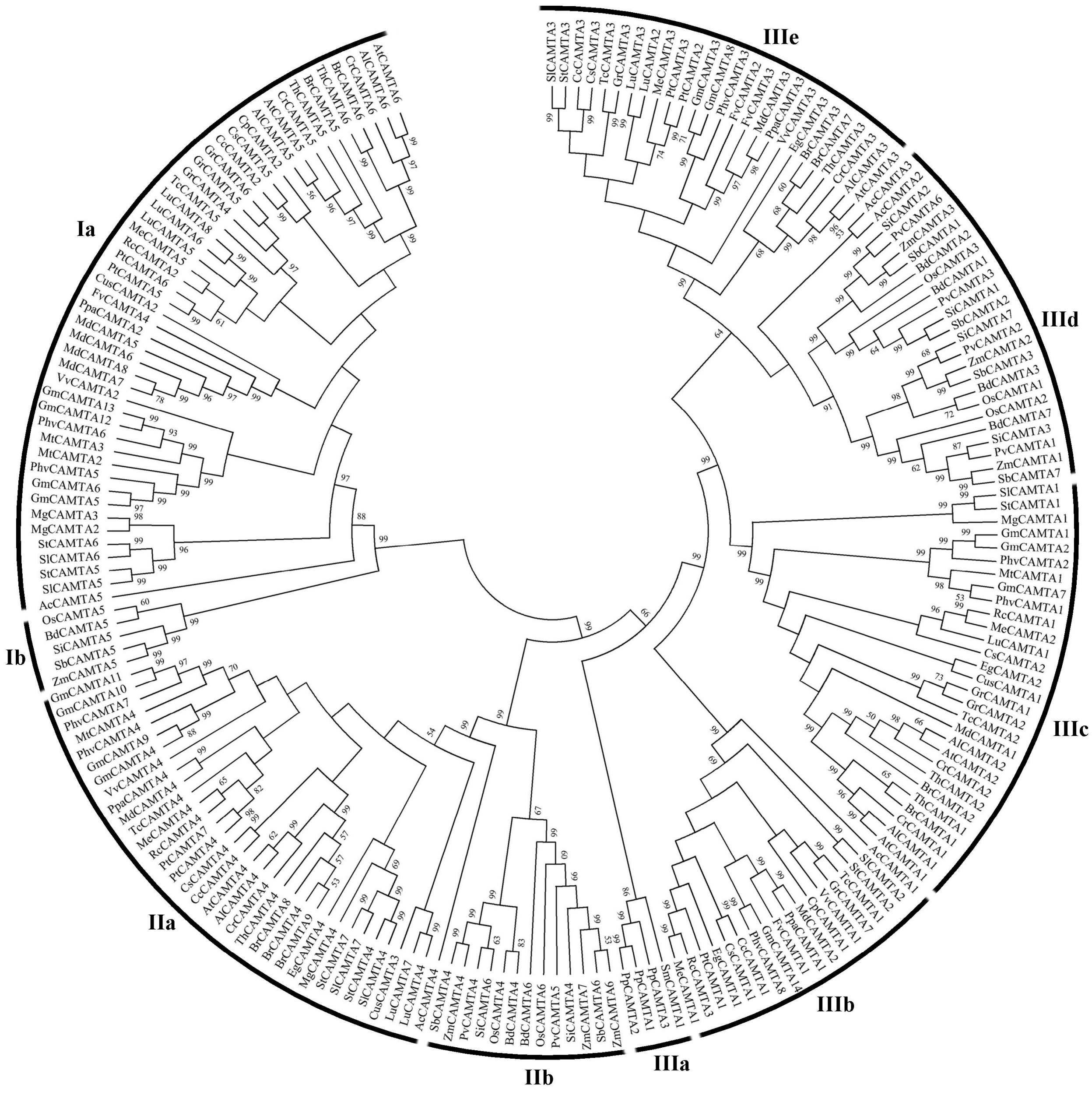

Figure S2 | The maximum likelihood (ML) phylogenetic tree of 200 plant CAMTA proteins identified in this study. The reconstruction percentage higher than 50% in the 1000 bootstrap analysis is indicated in the nodes.

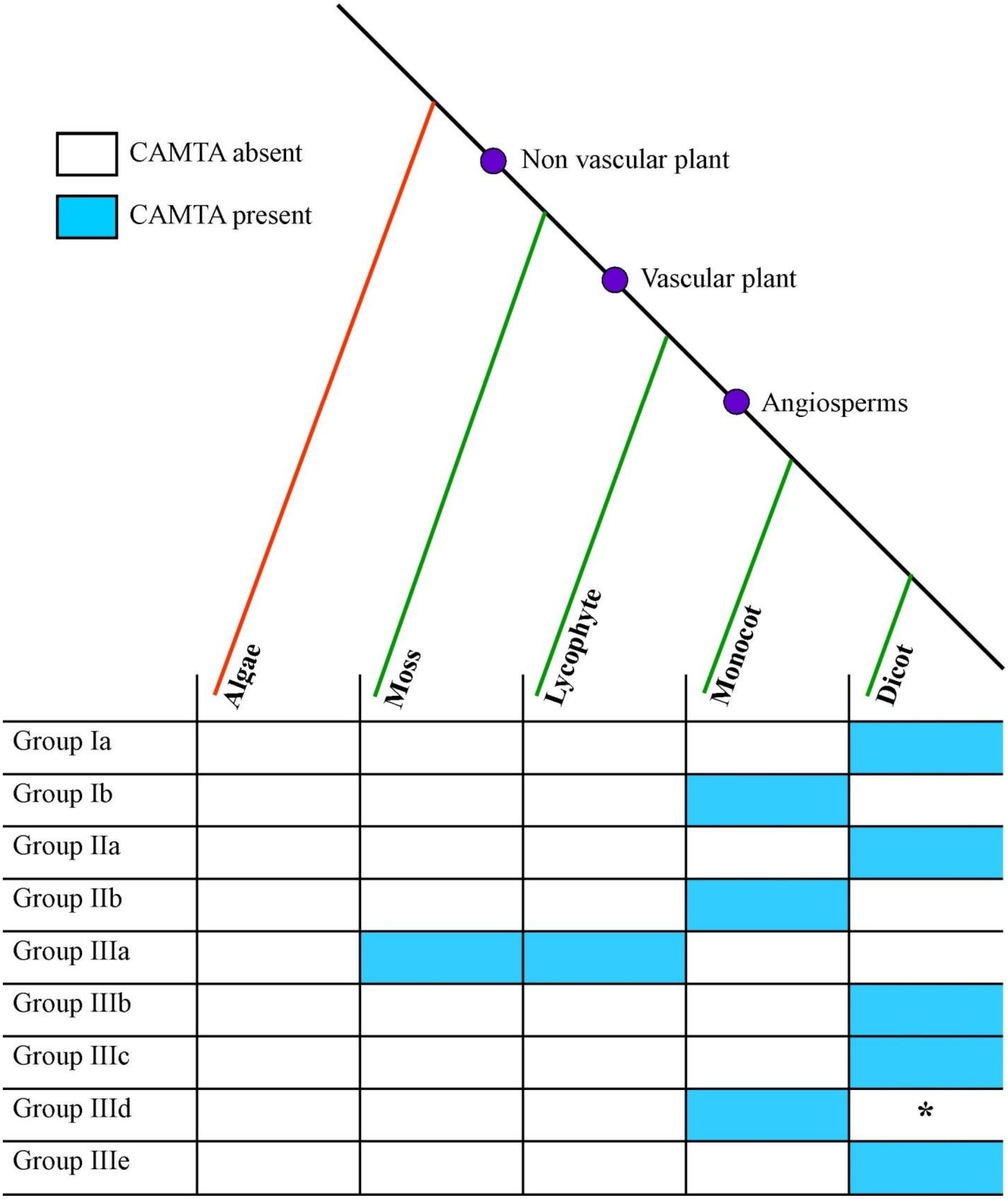

Figure S3 | Subgroup specific distribution of CAMTA proteins in different plant lineages. Existence of CAMTAs is indicated in blue and otherwise in white. The group IIId is dominated by monocot CAMTAs and contains only two dicot CAMTA genes from one species (AcCAMTA2 and 3), and thus is indicated as a “*”.

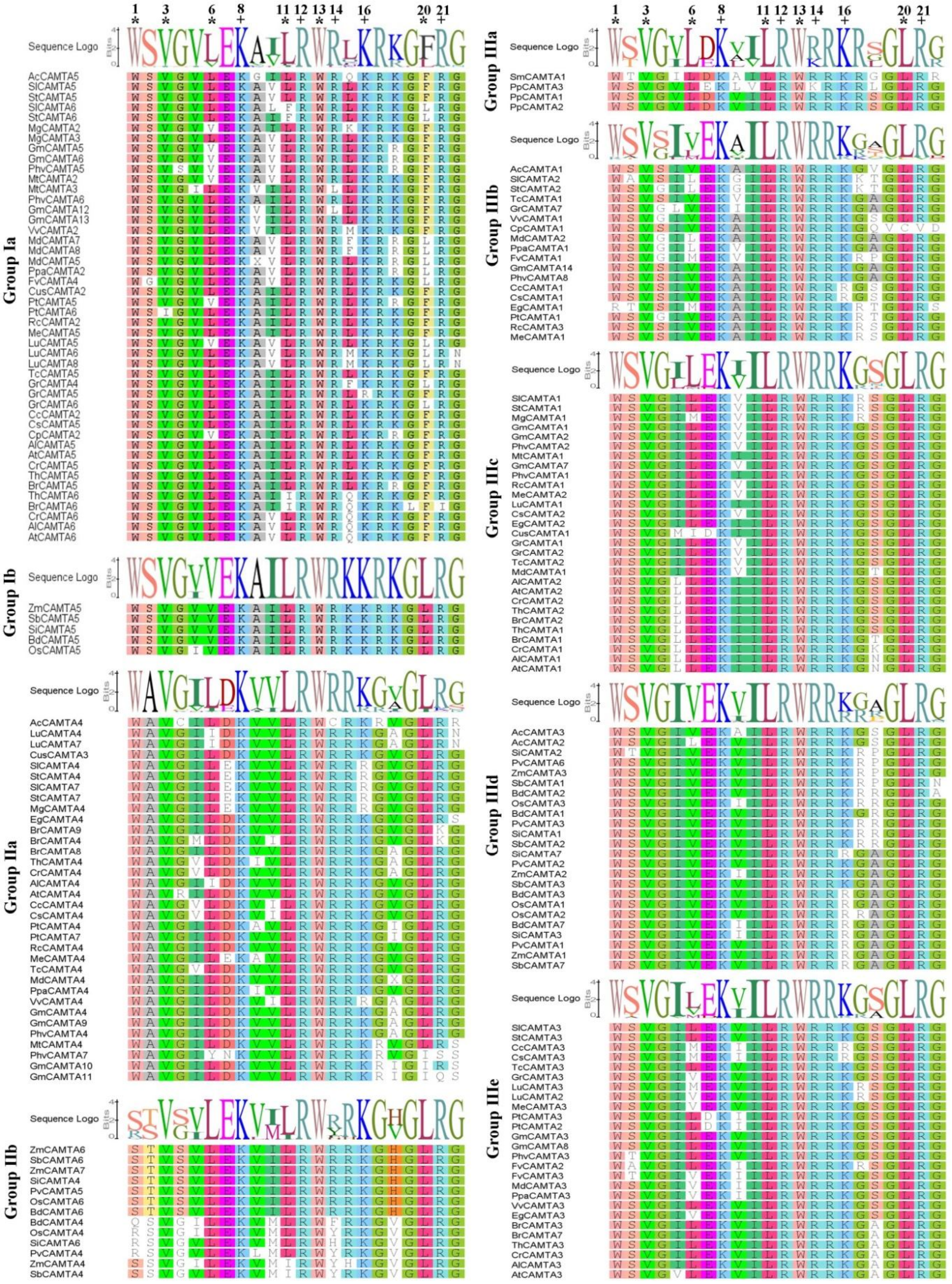

Figure S4 | Sequence logo of the CaMB domain of nine subgroups of CAMTA proteins. The “*” and “+” indicate conserved hydrophobic and positively charged residues in the CaMB domain.

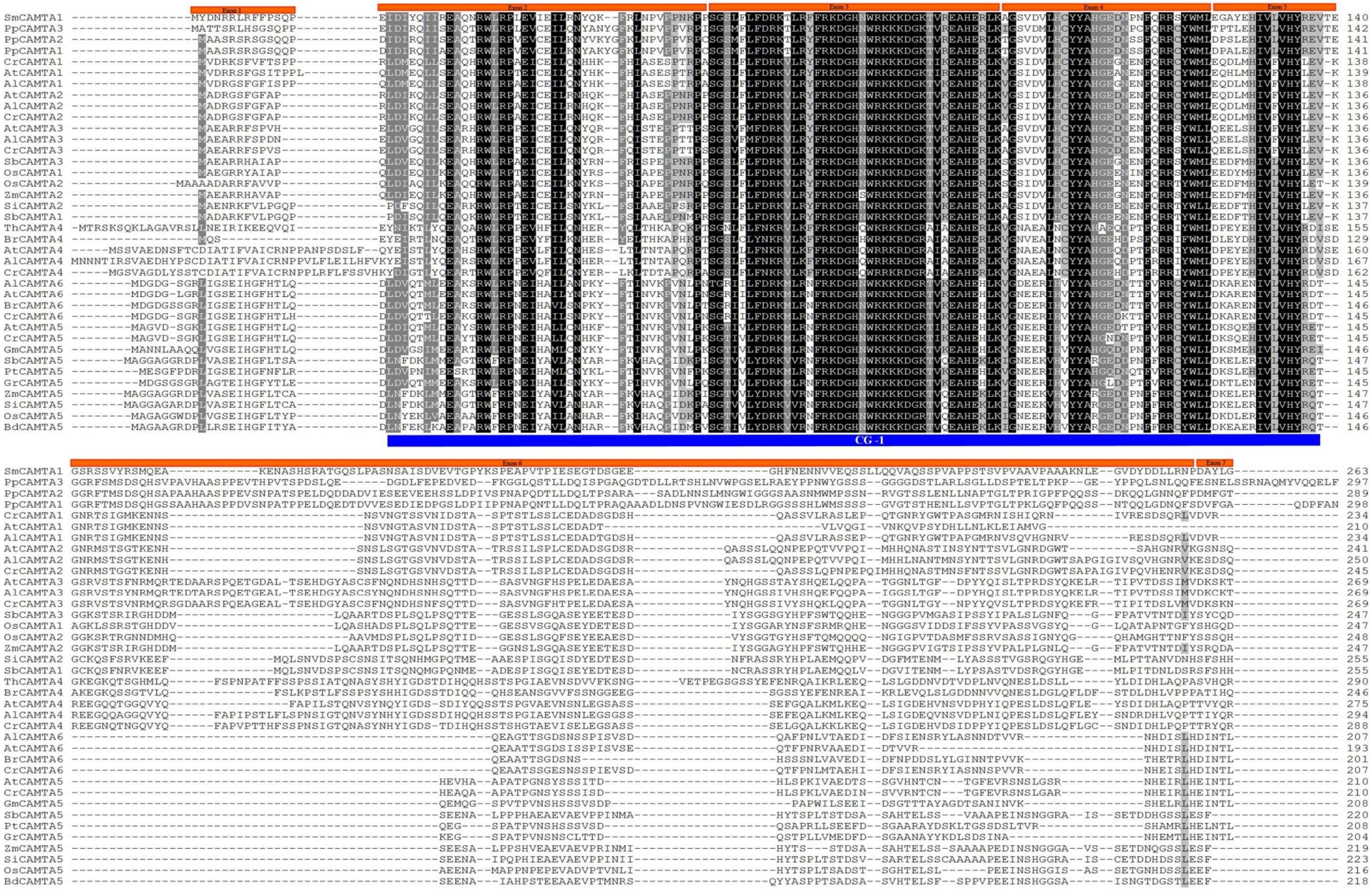
Exon 1
Exon 2
Exon 3
Exon 4
Exon 5
CG -1
Exon 6
Exon 7

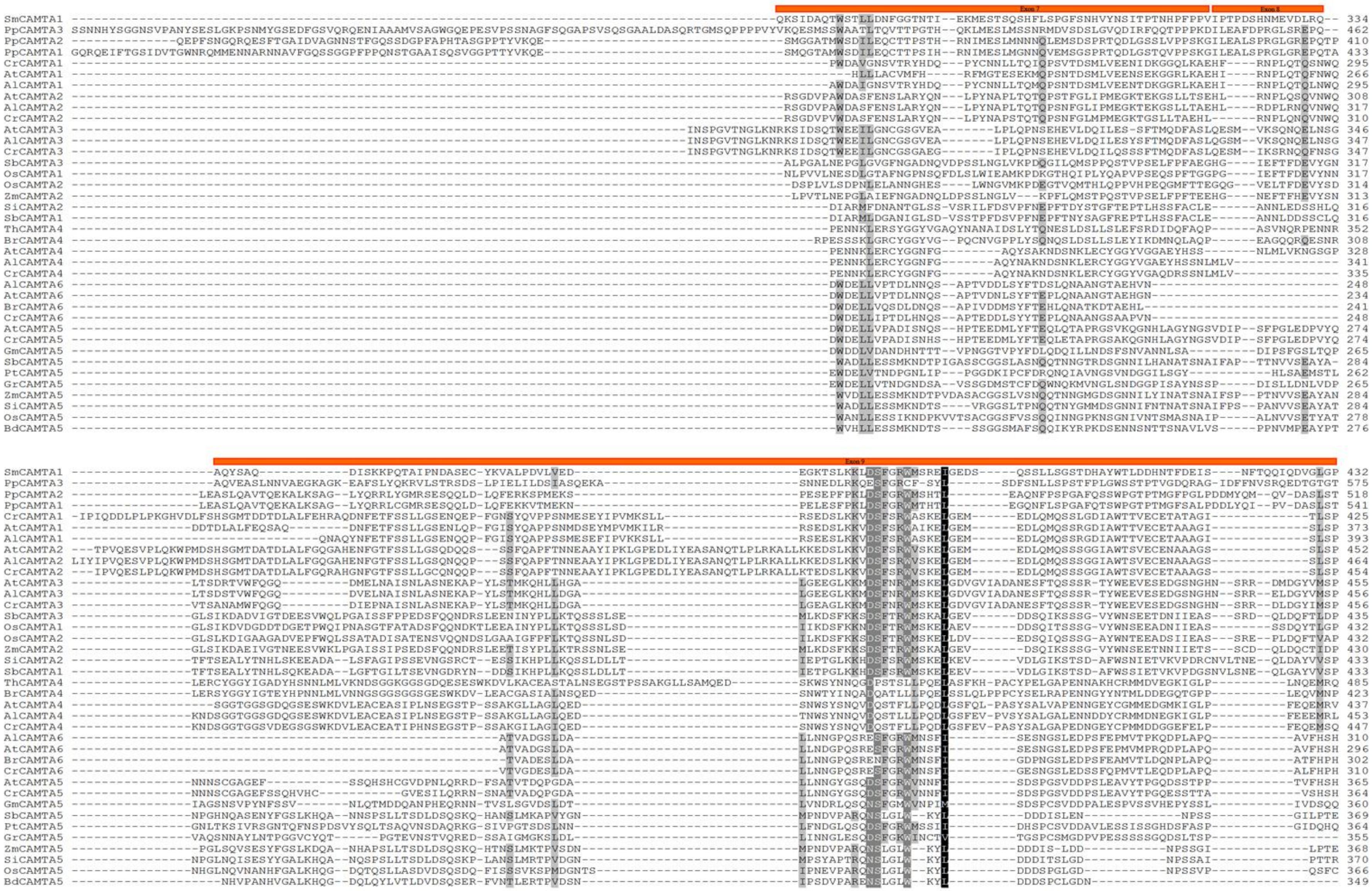

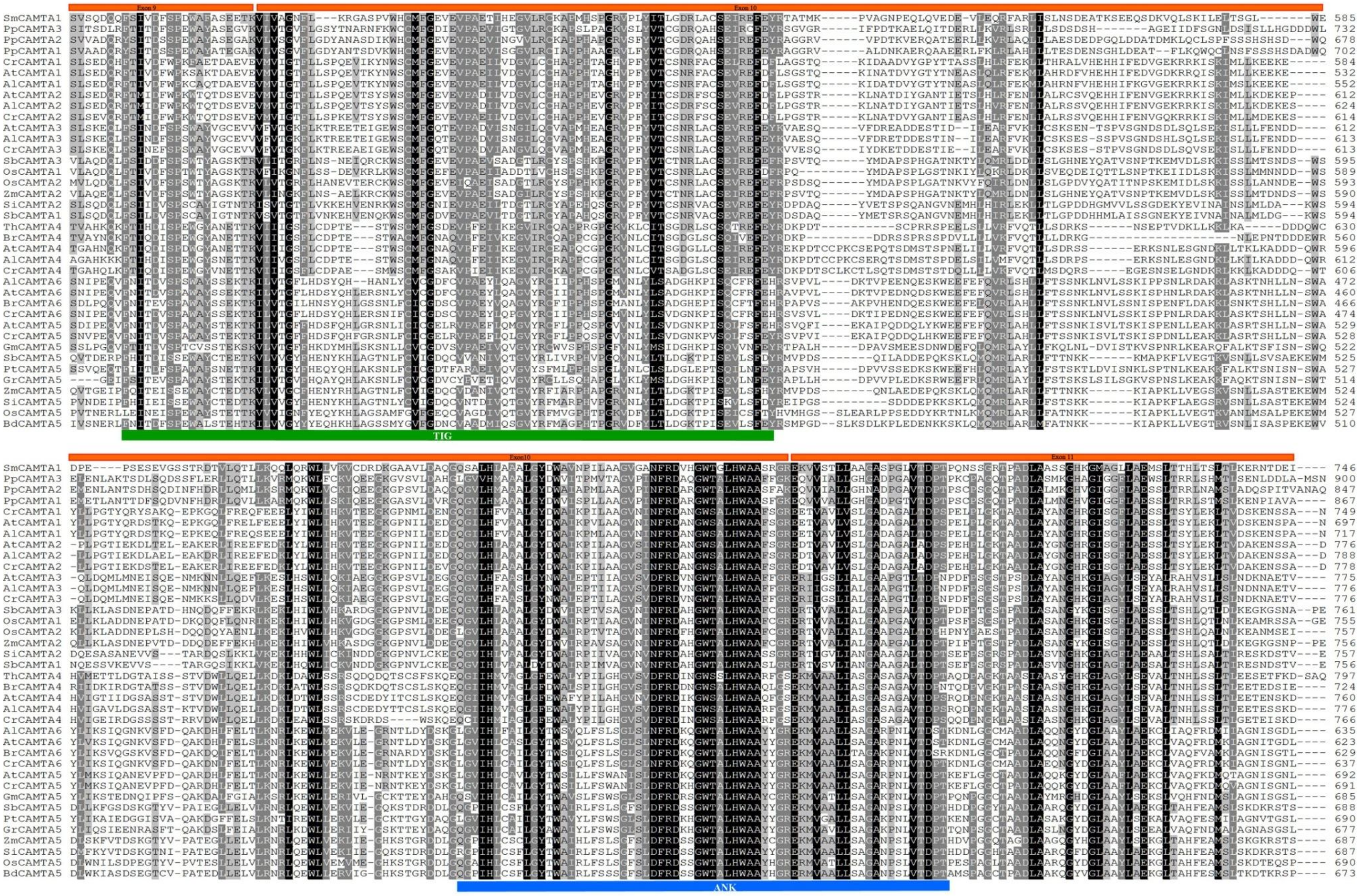
Exon 9
Exon 10
TIG
Exon10
Exon 11
ANK

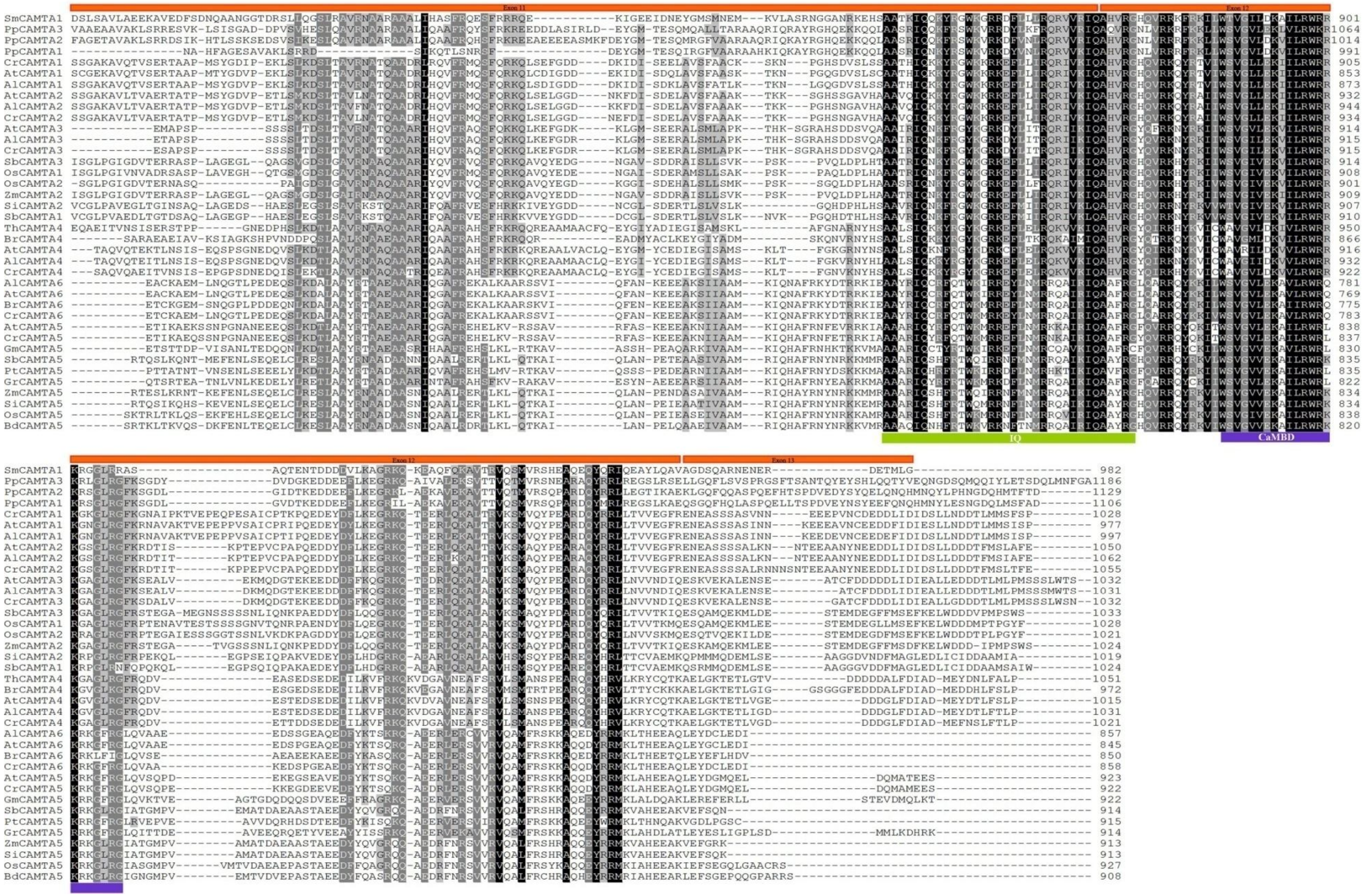

Figure S5 | Alignment of selected plant CAMTA proteins. Exons and domains are indicated above and below the alignment respectively.
